# Understanding the transfer reaction network behind the non-processive synthesis of low molecular weight levan catalyzed by *Bacillus subtilis* levansucrase

**DOI:** 10.1038/s41598-018-32872-7

**Published:** 2018-10-09

**Authors:** Enrique Raga-Carbajal, Agustín López-Munguía, Laura Alvarez, Clarita Olvera

**Affiliations:** 1 Departamento de Ingeniería Celular y Biocatálisis, Instituto de Biotecnología, UNAM. Av. Universidad #2001, Col. Chamilpa, C. P, 62210 Cuernavaca, Morelos Mexico; 2 Centro de Investigaciones Químicas-IICBA, Universidad Autónoma del Estado de Morelos. Av. Universidad #1001, Col. Chamilpa, C.P, 62210 Cuernavaca, Morelos Mexico

## Abstract

Under specific reaction conditions, levansucrase from *Bacillus subtilis* (SacB) catalyzes the synthesis of a low molecular weight levan through the non-processive elongation of a great number of intermediates. To deepen understanding of the polymer elongation mechanism, we conducted a meticulous examination of the fructooligosaccharide profile evolution during the levan synthesis. As a result, the formation of primary and secondary intermediates series in different reaction stages was observed. The origin of the series was identified through comparison with product profiles obtained in acceptor reactions employing levanbiose, blastose, 1-kestose, 6-kestose, and neo-kestose, and supported with the isolation and NMR analyses of some relevant products, demonstrating that all of them are inherent products during levan formation from sucrose. These results allowed to establish the network of fructosyl transfer reactions involved in the non-processive levan synthesis. Overall, our results reveal how the relaxed acceptor specificity of SacB during the initial steps of the synthesis is responsible for the formation of several levan series, which constitute the final low molecular weight levan distribution.

## Introduction

Fructans are fructosyl polymers produced by several plants and microorganisms whose biological function as energy reservoir and microbial defense agent has been demonstrated^1,2^. Depending on the linkage type, fructans can be classified as inulin, containing β(2-1) bonds in the main chain with β(2–6) ramifications; and levan, containing β(2–6) bonds in the main chain with β(2-1) ramifications^3^. Both fructans have interesting physicochemical and functional properties, and therefore a wide range of applications in the food and medical industries. Its low digestibility and potential to be degraded by probiotic bacteria made these polysaccharides an excellent prebiotic^3^. Besides this important application, they have been proposed as antioxidant, antiobesity, antiinflammatory, antitumoral, and antiviral agents^4–8^. They have also been studied due to their immunomodulatory, fibrinolytic and hypocholesterolemic activity^9,10^, among others. For this reason, there is a scientific interest in understanding the mode of action of the enzymes involved in fructan synthesis.

Fructansucrases (FSs) are glycosyltransferases that catalyze the synthesis of fructose-containing polymers from sucrose and are classified as levansucrases (EC 2.4.1.10) or inulosucrases (EC 2.4.1.9), depending on the type of synthetized polymer. *Bacillus subtilis* levansucrase (SacB), the most studied FS, consists of a 54 kDa single domain with a five-bladed-propeller fold that encloses a substrate binding central cavity^11^. In this site, the fructosyl moiety transfer takes place through a double-displacement reaction mechanism coordinated by three catalytic residues^12^. The mechanism involves breakage of the glycosidic linkage in a fructosyl donor (usually a sucrose molecule), resulting in the formation of a covalent fructosyl-enzyme intermediate and the release of the glucose moiety. Subsequently, the fructosyl moiety is transferred from the enzyme to an acceptor molecule; as a result, the acceptor is elongated in one fructosyl unit. The relaxed acceptor specificity of SacB has already been demonstrated through the evaluation of several molecules as fructosyl acceptors in SacB reactions using sucrose as donor substrate, such as alcohols (xylitol, glycerol), monosaccharides (glucose, fructose, mannose, xylose, galactose, arabinose, fucose), disaccharides (isomaltose, maltose, melibiose, cellobiose, lactose, maltulose) and oligosaccharides (maltooligosaccharides, oligolevans)^13–15^. When a water molecule acts as acceptor of the fructosyl intermediate, fructose is released into the reaction media (hydrolysis). As thoroughly demonstrated, the hydrolysis/transfructosylation specificity is strongly defined by reaction conditions^16–18^.

In reactions with sucrose as the single substrate, SacB catalyzes the formation of polymeric products and produces two distributions: a high molecular weight (HMW) and a low-molecular weight (LMW) levan with an average molecular weight of 2300 kDa and 7.2 kDa, respectively^19^. Although levan is the major product in many levansucrase (LS) reactions, fructooligosaccharides (FOS) have been associated to the polymer synthesis since the very first reports dealing with LS reaction mechanism, half a century ago^20,21^. Nevertheless, up to now only a few FOS have been identified, mainly due to analytical limitations. Nowadays, high-performance techniques, such as anion exchange chromatography with pulsed amperometric detection (HPAE-PAD), have allowed the resolution and identification of some oligosaccharides in reactions with different LSs^17,22–24^. Recently, the observation of reaction intermediates using this type of analysis allowed us to elucidate the SacB levan elongation mechanisms; namely, a processive and a non-processive mechanism for HMW and LMW levan synthesis respectively^19^. In effect, SacB synthesizes the LMW levan through sequential fructosyl capture, transfer and release of the fructosylated intermediates, i.e. FOS of an increasing degree of polymerization. As a consequence of this elongation mechanism, the final product consists of a large number of intermediates and final elongation products, actually a distribution of “levan molecules”, most of them still not identified. Most importantly, the mechanistic and structural bases of this biosynthetic transformation remain unclear.

In order to gain a better understanding about the mode of action of SacB levansucrase in the non-processive elongation of levans, we meticulously examined the synthesis products following the formation and evolution of intermediates by HPAE-PAD not only in reactions with sucrose as a single substrate but also in the presence of different inherent acceptors of the reaction. This strategy allowed the identification of several polymer chain initiators, as well as intermediate fructan series, which were substantiated by NMR analyses of some intermediates, all associated to levan synthesis. From a more general perspective, our results illustrate how the initial broad acceptor specificity of a single enzyme results in the formation of a heterogeneous distribution of polysaccharide chains.

## Results and Discussion

### Distinct intermediate series emerge throughout the non-processive synthesis of levans

**Figure 1:**
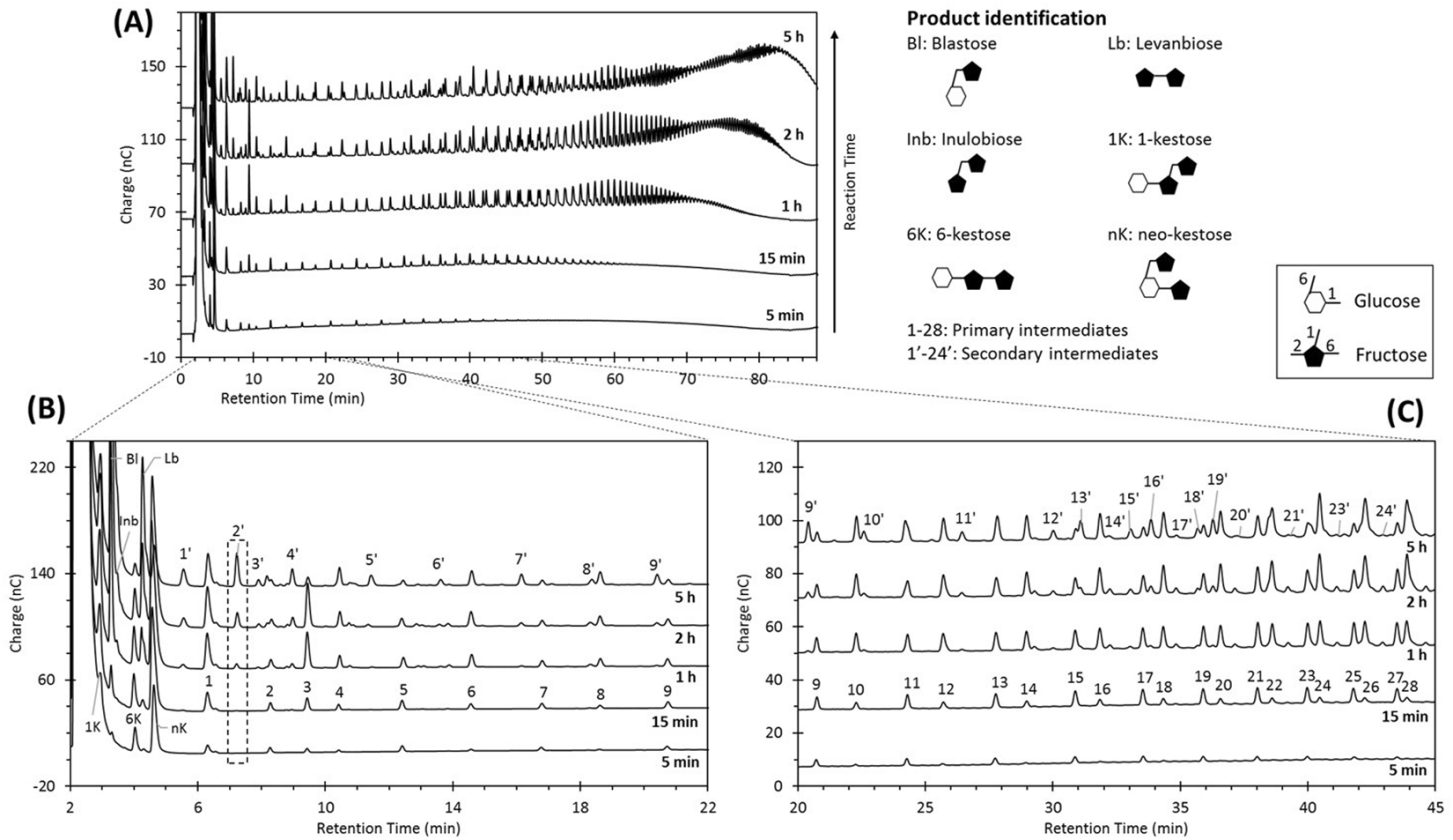
Evolution of the non-processive synthesis of levans followed by HPAE-PAD analysis. B and C panels are amplified views of chromatogram A. Unidentified products were classified as primary intermediates (1–28) and secondary intermediates (1′-24′). The first are produced early in the reaction, whereas the latter appear after 1 hour (e.g. peak 2′). Reaction conditions: SacB 0.5 µM, sucrose 100 g L^−1^, pH 6, 25 °C.

As depicted in Fig. 1A, SacB synthesizes a distribution of LMW levans through the elongation of a wide variety of intermediates, whose size increases over time during the reaction. Actually, the spectrum of intermediates and final products includes more than 150 compounds. Besides glucose and fructose, products of the transferase and hydrolysis reactions, the comparison with standards (Supplementary Fig. S1) allowed the identification of disaccharides such as: blastose [β-D-Fru*f*-(2 → 6)-D-Glc*p*], levanbiose [D-Fru*f*-(2 → 6)-β-D-Fru*f*], and inulobiose [β-D-Fru*f*-(2 → 1)-β-D-Fru*f*], as well as the trisaccharides: 1-kestose [β-D-Fru*f*-(2 → 1)-β-D-Fru*f*-(2 ↔ 1)-α-D-Glc*p*], 6-kestose [β-D-Fru*f*-(2 → 6)-β-D-Fru*f*-(2 ↔ 1)-α-D-Glc*p*], and neo-kestose [β-D-Fru*f*-(2 → 6)-α-D-Glc*p*-(1 ↔ 2)-β-D-Fru*f*] as products of transferase activity to sucrose (Fig. 1B).

**Figure 2:**
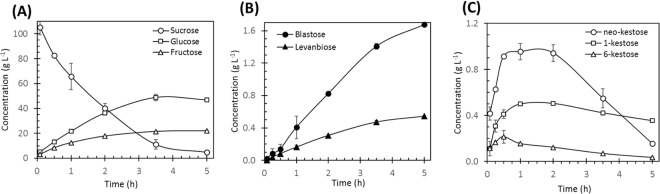
Formation kinetics of monosaccharides, disaccharides and trisaccharides during the non-processive synthesis of levans by SacB. Reaction conditions: SacB 0.5 µM, sucrose 100 g L^−1^, pH 6, 25 °C.

In a more detailed examination of the fructooligosaccharides profile, we can observe two distinct series of intermediates in the same molecular weight range (Fig. 1B and C). One of this intermediates group is synthesized from the very beginning of the reaction and is conserved until the end of the reaction, whereas a second group of intermediates emerge after 1 hour of reaction (as example, see the box for peak 2′ in Fig. 1B). The formation of different fructooligosaccarides series may be associated with the elongation of different initiators in the synthesis levan process. As deduced from the synthesis, accumulation and consumption of oligosaccharides described in both Figs 1 and 2, the non-processive synthesis of LMW levan may be summarized in three stages or phases as follows:

#### Early phase

During the first 30 minutes of reaction less than 20% of sucrose is converted into glucose (byproduct of both transfructosylation and hydrolysis reactions), fructose (product of hydrolysis) and various transfructosylation products. Among the products of transfer to sucrose, three main compounds are formed and accumulated from the very beginning of the reaction: 1-kestose, 6-kestose and neo-kestose (Fig. 1B). Neo-kestose accumulates and remains as the major trisaccharide (up to 0.9 g L^−1^), twice the concentration of 1-kestose and 4.5 times that of 6-kestose (Fig. 2C). At the same time, the first group of products, which we define as “primary intermediates”, appears in solution increasing in size over time, reaching DPs of approximately 30. The first 28 primary intermediates of the early phase are numbered in the 15-min oligosaccharide profile shown in Fig. 1B and C.

#### Late phase

As the reaction proceeds, new products are observed when more than 20% of sucrose is converted. Among these products, the disaccharides blastose and levanbiose reach 0.4 and 0.16 g L^−1^ after one hour of reaction and duplicate their concentration one hour later (Fig. 2B), while inulobiose is barely detected (Fig. 1B). The formation of these three products is related to the rapid accumulation of glucose and fructose, which reach 36 and 18 g L^−1^, respectively, when sucrose conversion approaches 60%, after 2 h of reaction (Fig. 2A). The most important finding in this phase is the identification of an additional group of intermediates, designed as “secondary intermediates” and observed after one hour of reaction (Fig. 1B and C). These secondary intermediates include FOS of DP 3 to approximately 20, in contrast to primary intermediates that, at this reaction time, reach DPs higher than 40. During the late phase, neo-kestose concentration remains constant, whereas 1-kestose and 6-kestose reach maximum concentrations of 0.5 g L^−1^ and 0.21 g L^−1^ (Fig. 2C).

#### Sucrose depletion phase

The final reaction stage occurs when sucrose is depleted, resulting in the LMW distribution of levan, with an average DP of 50^19^, containing both primary and secondary intermediates, as well as their elongation products. It is important to highlight the decrease of neo-kestose when the sucrose conversion reaches more than 60% (Fig. 2C). In this third stage of the reaction, 1-kestose is the most abundant trisaccharide (0.35 g L^−1^), whereas 6-kestose has decreased to 0.15 g L^−1^ (Fig. 2C). Meanwhile, blastose and levanbiose concentration increase during this stage of the reaction, reaching 1.67 and 0.54 g L^−1^ respectively (Fig. 2B).

As described so far, SacB synthetizes two families of intermediates during the early and late phases of reaction. To ascertain the origin and the chemical nature of these intermediate groups, we carried out reactions using sucrose as fructosyl donor and employing intermediates inherent to the reaction as acceptors, namely 1-kestose, 6-kestose, neo-kestose, blastose and levanbiose.

### Primary intermediates: levan series from 1-kestose and 6-kestose

**Figure 3:**
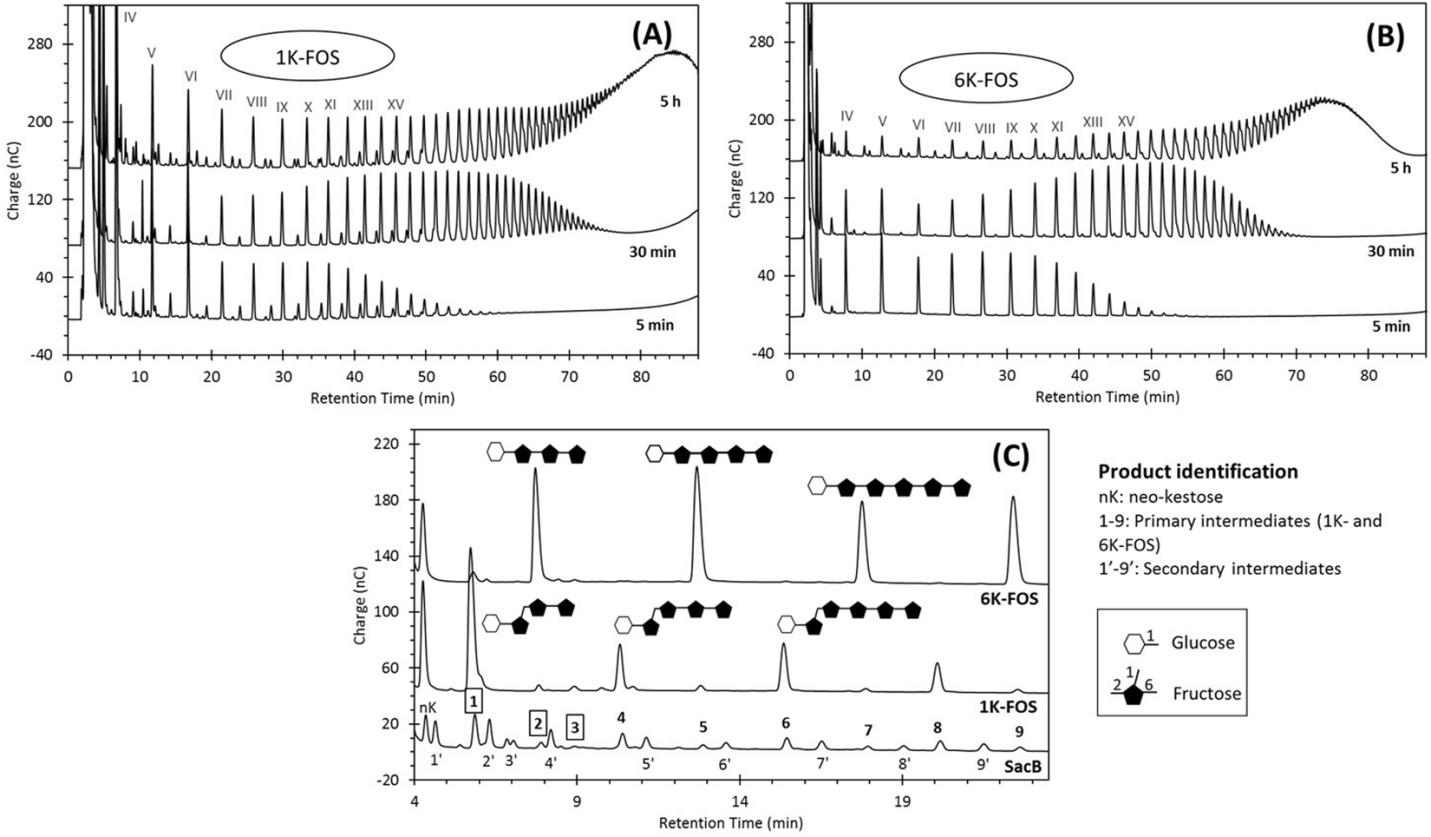
Evolution of the SacB-sucrose/acceptor reactions, using (**A**) 1-kestose and (**B**) 6-kestose as acceptor, analyzed by HPAE-PAD. Roman numerals indicate the estimated DP of the oligosaccharide along each oligosaccharide series. (**C**) Comparison of the 5-min product profile of (**A**) and (**B**) with the final product profile of the SacB-sucrose reaction (line ‘SacB’). Purified primary intermediates are marked with framed numbers. Product structures are proposed according to NMR analyses. Reaction conditions: 0.5 µM SacB, 292 mM sucrose, 5 mM acceptor.

As shown above, 1-kestose, 6-kestose and neo-kestose are the first transfructosylation products. The structural difference between these products is an evidence of the low enzyme regioselectivity in the first fructosyl transfer event, in which sucrose acts as both donor and acceptor molecule. The primary intermediates observed in the early reaction phase seem to originate from these three trisaccharides. To demonstrate this hypothesis, SacB-sucrose/acceptor reactions were performed using 1-kestose or 6-kestose as acceptors. In Fig. 3A it is observed that in the SacB-sucrose/1-kestose reaction the synthesis of one oligosaccharide series is favored, but the final products profile is similar to that in SacB-sucrose reactions, including primary and secondary intermediates. A similar behavior is observed for SacB-sucrose/6-kestose reactions, with the synthesis of a clearly different oligosaccharide series, present since the beginning of the reaction (Fig. 3B). We can therefore conclude that both trisaccharides are effectively elongated by SacB, leading to the formation of two oligosaccharide series: 1-kesto-fructooligosaccharides (1K-FOS) and 6-kesto-fructooligosaccharides (6K-FOS), with more than 15 compounds integrating each series. The estimated DP of the detected oligosaccharides is indicated as a roman numeral in Fig. 3A and B.

Finally, Fig. 3C shows a products profile comparison between the 1K-FOS, 6K-FOS and SacB-sucrose products. For the sake of clarity, the comparison focuses only on the small intermediates of each series, with retention time of less than 24 min. It may be noticed that 1K-FOS with DP from 4 to 7 correspond to the primary intermediates 1, 4, 6, and 8; whereas the 6K-FOS with the same DP coincide with the primary intermediates 2, 5, 7, and 9. Considering that the components of both series elute at different times in the chromatographic separation, also different from the inulin-type FOS series used as standard (see Supplementary Fig. S1), and that at no time during the reaction phases 1-nystose was detected, we suggest that the fructosyl transfer to the trisaccharides involves elongation solely through β2–6 bonds. In order to corroborate this, some of the primary intermediates in SacB reaction (peaks 1 and 2) were isolated by means of SEC and rp-HPLC, and characterized by 1D and 2D NMR spectral analysis. Peak 1 was identified as 1,6-nystose [β-D-Fru*f*-(2 → 6)-β-D-Fru*f*-(2 → 1)-β-D-Fru*f*-(2 ↔ 1)-α-D-Glc*p*], and peak 2 as 6,6-nystose [β-D-Fru*f*-(2 → 6)-β-D-Fru*f*-(2 → 6)-β-D-Fru*f*-(2 ↔ 1)-α-D-Glc*p*] (Supplementary Figs S2 and S3). These tetrasaccharides are products of the fructosyl transfer at position 6 of the terminal fructosyl moiety of 1-kestose and 6-kestose, respectively. As 1K-FOS and 6K-FOS series cover the family of primary intermediates, except for peak 3, these results support the idea that 1-kestose and 6-kestose are the main inherent initiators during the early phase of LMW levan synthesis.

The acceptor nature of 1-kestose and 6-kestose has already been documented both with SacB as well as with LSs from *Aerobacter levanicum* and *Bacillus natto*. In early works, Feingold *et al*.^20^ reported the isolation of FOS from sucrose-growing cells as well as from cell-free levansucrase extracts of *A. levanicum*, with 1-kestose as the major product and traces of 6-kestose. Moreover, these authors reported the production of up to three fructosides from successive transfers to 1-kestose, and a tetrasaccharide obtained from the transfer to 6-kestose. Later, Rapoport *et al*.^25^ reported 1-kestose as the first product from *B. subtilis* levansucrase reactions, establishing that larger products can be obtained by fructosyl transfers to 1-kestose in β2–6 position. More recently, Iizuka *et al*.^26^ found that 1-kestose, 6-kestose and, to a lesser extent neo-kestose, are initiators of levan synthesis reactions by LS from *B. natto*, as demonstrated by the presence of these compounds in levan structures through nuclear magnetic resonance analysis. Our results show for the first time the chromatographic identification of the 1K-FOS and 6K-FOS levan series, with more than 15 members, all of them intrinsic products of the non-processive synthesis of LMW levan by *B. subtilis* levansucrase.

### Secondary intermediates: synthesis of blasto-fructooligosaccharides and oligolevans

As LMW levan synthesis takes place, additional intermediates are observed. Consequently, these intermediates must have their origin in alternative initiators also formed during the reaction. In effect, as already shown in Figs 1 and 2, during the LMW levan synthesis the accumulation of levanbiose, inulobiose and blastose was observed. The origin of the difructosides is associated to SacB-catalyzed fructosyl transfers to fructose molecules, with levanbiose (β2–6 bond) as the preferential product, and inulobiose (β2-1 bond) formed to a lesser extent. On the other hand, the origin of blastose is less obvious, but usually related to the hydrolysis product of neo-kestose^27^. Nevertheless, its formation through the direct fructose transfer to glucose should also be considered^20^.

**Figure 4:**
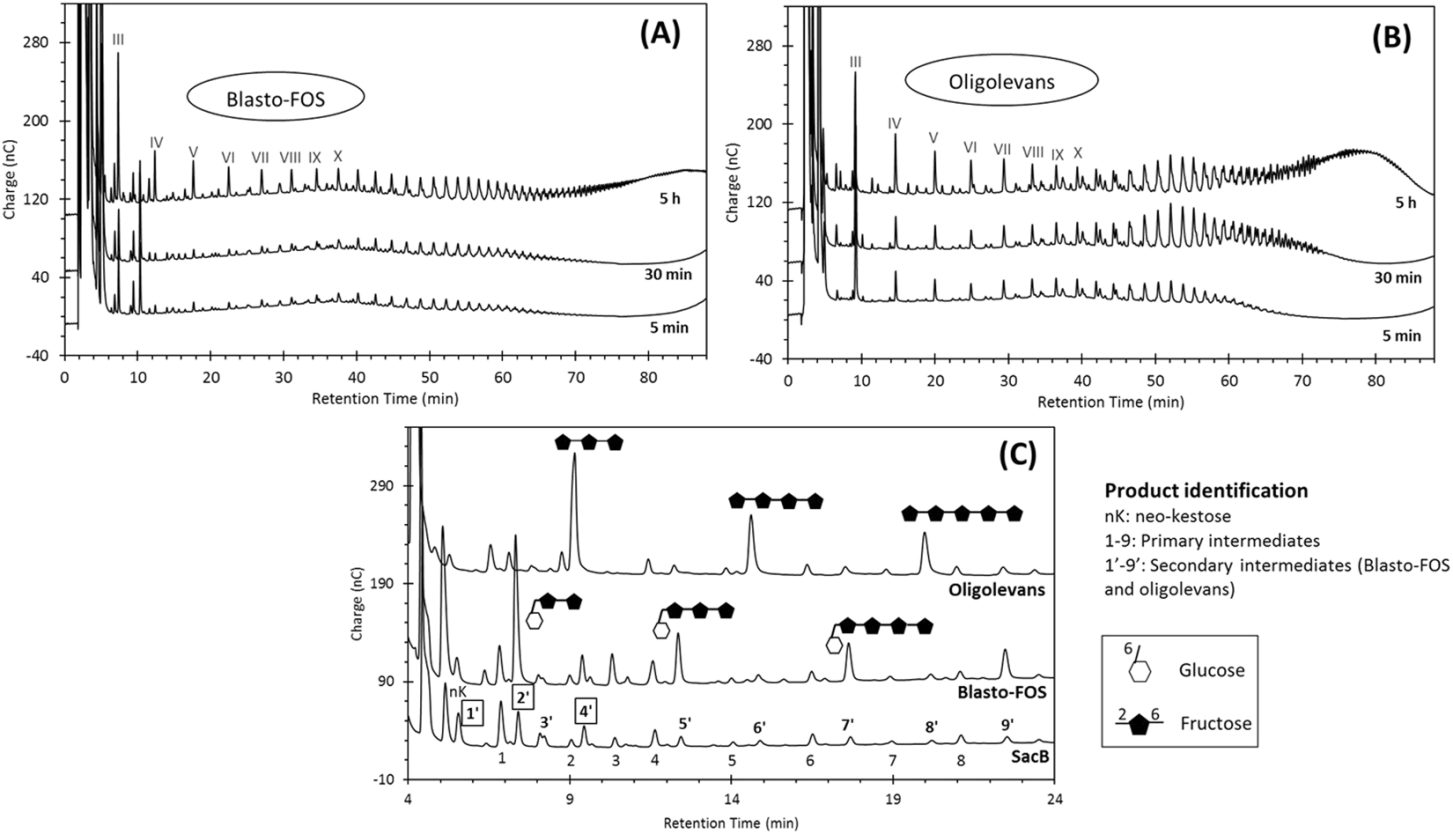
Evolution of the SacB-sucrose/acceptor reactions, using (**A**) blastose and (**B**) levanbiose as acceptor, analyzed by HPAE-PAD. Roman numerals indicate the estimated DP of the oligosaccharide along each oligosaccharide series. (**C**) Comparison of the final product profile of (**A**) and (**B**) with the final profile of the SacB reaction (‘line SacB’). Purified secondary intermediates are marked with framed numbers. Product structures are proposed according to NMR analyses. Reaction conditions: 0.5 µM SacB, 292 mM sucrose, 20 mM acceptor.

Bearing in mind that blastose and levanbiose are the major low-DP products in the final product profile of SacB-catalyzed reaction, both disaccharides were tested as acceptors to elucidate their role in the formation of intermediates observed during the LMW levan synthesis, either in the primary or secondary groups. As in the initial assays, we observed a lower affinity of SacB for these acceptors, as their concentration had to be increased up to 20 mM in order to detect the reaction products. The evolution of the product profile during the SacB-sucrose/blastose reaction is shown in Fig. 4A. The oligosaccharide marked with the roman numeral III (most probably a DP 3 product) is initially produced, followed by the synthesis of an oligosaccharide series that stand out among the other final products. The oligosaccharides within this series are presumably the result of subsequent β2–6 fructose transfers to blastose, constituting a novel FOS series that we propose to name “blasto-fructooligosaccharides” (Blasto-FOS). Most importantly, this series correspond to some of the secondary intermediates already described, as observed in the comparison with the SacB product profile (Fig. 4C). With the aim to corroborate the structure of this blasto-FOS series, peak 2′ was isolated and characterized by 1D and 2D NMR. In the ^1^H NMR spectrum, two anomeric protons were observed at δ_H_ 5.27 (d, *J* = 3.76 Hz) and 4.67 (d, *J* = 7.7 Hz), the coupling constant of these signals evidenced that this compound was a mixture of α-orientated and β-orientated glucoside isomers, in a ratio of 27:100 according to signal integration. HSQC showed their attachment to the anomeric carbons at δ_C_ 94.72 and 98.56, respectively. Besides, suggesting that C-1 position of the glucose moiety was free. Moreover, ^13^C NMR spectrum showed the presence of other two quaternary anomeric carbon signals at δ 106.56, and 106.39, attributed to two fructosyl residues. The connectivity was unravelled from key crosspeaks in the HMBC spectra, which exhibited a correlation of the quaternary carbon atom (C-2′) at δ_C_ 106.56 of Fru′ with the methylene protons (H-6) of Glc, as well as a correlation of the second quaternary proton (C-2″) at δ_C_ 106.39 of Fru′′ with the methylene protons of H-6′ (δ_H_ 3.99) of Fru′. Accordingly, peak 2′ was characterized as the isomeric mixture 27/100 β-D-Fru*f*-(2 → 6)-β-D-Fru*f*-(2 → 6)-α/β-D-Glc*p*, here named “blastotriose”, a product derived from a single fructosyl transfer at position 6 of the fructosyl moiety of the sucrose isomer blastose (Supplementary Fig. S4).

Following the same rational, an additional oligosaccharide series was synthesized using levanobiose as acceptor (Fig. 4B). These products most probably correspond to oligolevans, FOS composed of β2–6 linkages lacking a terminal glucosyl moiety. At least eight oligolevans with DP from 3 to 10 can be clearly identified. The estimated DP of each product is indicated with roman numerals in Fig. 4B. The comparison with the SacB-sucrose reaction profile shows that this series correspond with some of the secondary intermediates, such as peaks 4′, 6′, and 8′ (Fig. 4C). This was confirmed through the purification and characterization of peak 4′, identified by NMR as levantriose [β-D-Fru*f*-(2 → 6)-β-D-Fru*f*-(2 → 6)-β-D-Fru*f*], the trisaccharide into the oligolevans series (Supplementary Fig. S5).

In addition, we were able to purify peak 1′, a secondary intermediate not aligned with already described series (Fig. 4C). The enzymatic hydrolysis of this product with Fructozyme, an enzymatic preparation containing endo- and exo-inulinases, rendered equal amounts of fructose and glucose (data not shown). The 1D and 2D NMR analyses of peak 1′displayed one anomeric proton signal at δ_H_ 5.44 (d, *J* = 2.9 Hz), assigned to an α-oriented glucosyl moiety. HSQC showed its attachment to the anomeric carbon at δ_C_ 94.57. In addition, ^13^C NMR spectrum evidenced the presence of one quaternary anomeric carbon arising from a fructosyl unit. From the analysis of the signals present in the COSY, HSQC, and HMBC spectral data, the full assignment of the ^1^H and ^13^C signals belonging to the two spin systems was achieved. The interglycosidic linkage of the two sugar units was derived from the HMBC correlations between H-2 of glucose at δ_H_ 3.80 and C-2 of fructose at δ_C_ 106.13, indicating that this disaccharide has a reducing glucose. The NMR data allowed identifying peak 1′ as the sucrose isomer β-D-Fru*f*-(2 → 2)-α-D-Glc*p*, called in this work “Ercose”, a product most probably derived from the transfructosylation of glucose.

There are several reports in the literature dealing with the possibility of a fructosyl moiety transfer from sucrose to fructose or glucose by levansucrases. For instance, levanbiose, inulobiose, blastose, ercose and β-D-fructofuranosyl-(2 → 3)-D-glucose were found among the transfer products of the LS from *A. levanicum*^20^. In the same context, the incorporation of added radiolabeled fructose into oligolevans and levans was also reported in SacB reactions with sucrose^21^. However, this is the first report providing evidence of the blastose role as acceptor in levan synthesis. It is important to mention that the acceptor role of fructose and levanbiose has only been observed in reactions with high acceptor concentrations^21,26,28^. During the LMW levan synthesis reaction high levels of hydrolysis (H) are initially observed, while the transferase (T) activity increases towards the end of the reaction. For instance, at 292 mM sucrose the H/T ratio decreases from 62/28 to 47/53 during the reaction; consequently, fructose accumulates as a final product up to 22 g L^−1^ with evidence of the formation of oligolevans when less than 8 g L^−1^ of fructose has accumulated, as shown in Fig. 2A. As previously suggested, the hydrolytic activity of the levansucrase and the formation of potentially acceptor fructose molecules are additional factors influencing the composition of the final LMW levan distribution^29^.

### Neo-FOS are precursors of blasto-FOS

**Figure 5:**
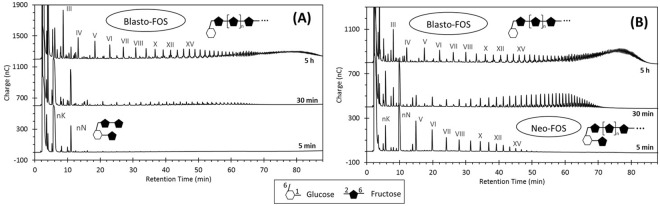
Evolution of the SacB-sucrose/acceptor reaction employing (**A**) neo-kestose (nK) and (**B**) 6-neo-nystose (nN) as acceptor, analyzed by HPAE-PAD. Roman numerals indicate the estimated DP of the oligosaccharide along each oligosaccharide series. Reaction conditions: 0.5 µM SacB, 292 mM sucrose, 20 mM neo-kestose or 5 mM 6-neo-nystose.

Finally, we investigated the role of neo-kestose in levan synthesis, carrying out the corresponding acceptor reaction. It is important to point out that, as in the case of blastose and levanbiose, neo-kestose is required in large concentrations (20 mM) to obtain significant amounts of the products. As depicted in Fig. 5A, in this reaction one product is markedly produced within the first 5 minutes of reaction. This product corresponds to the primary intermediate number 3 in Fig. 1B, most likely the product of a single β2–6 fructosyl transfer to neo-kestose. Peak 3 was successfully isolated and NMR data allowed to identify it as β-D-Fru*f*(2 → 6)-β-D-Fru*f*-(2 → 6)-α-D-Gl*p*-(1 ↔ 2)-β-D-Fru*p*, here named “6-neo-nystose” (Supplementary Fig. S7).

Towards the end of the reaction using neo-kestose as acceptor, the blasto-FOS series dominates above the other products. The late formation of this series is concurrent with the consumption of neo-kestose and 6-neo-nystose towards the end of the reaction. The depletion of these two neo-FOS is similarly observed at the final stage of the LMW levan synthesis, as shown in Fig. 1A for 6-neo-nystose (peak 3) and neo-kestose in Fig. 2C.

To examine the role of other member of the levan neo-series, the tetrasaccharide 6-neo-nystose was assayed as acceptor (Fig. 5B). We found that 6-neo-nystose favors with high affinity the formation of a completely new oligosaccharide series, labeled with roman numerals in the 5-min product profile shown in Fig. 5B, and most likely consisting of levan-type neo-FOS produced by successive transfers to 6-neo-nystose reaching a DP larger than 15. The presence of a new complete series derived from 6-neo-nystose, but not from neo-kestose reflects the higher acceptor specificity of the tetrasaccharide and is consistent with the high concentration of neo-kestose (20 mM) needed to observe an acceptor reaction in comparison with the lower amounts of 6-neo-nystose (5 mM) required in the reactions. However, this new series is gradually consumed as the reaction proceeds, resulting in the accumulation of blasto-FOS, which are depicted in Fig. 5B, marked with roman numerals.

SacB-catalyzed reactions with neo-kestose or 6-neo-nystose as sole substrates yielded mainly hydrolysis products: fructose and blastose from neo-kestose, and fructose and blastotriose (trisaccharide in the blasto-FOS series) from 6-neo-nystose, revealing that the two substrates may also be used by SacB as substrate fructosyl donors. Nevertheless, transfructosylation products were also observed during the initial steps in both reactions, revealing that they may also be used by SacB as substrate fructosyl donors (Supplementary Figs S8 and S9). This result is relevant as other transfructosylation products such as blastose, levanbiose, 1-kestose or 6-kestose did not serve as donor substrates, as no products could be detected upon incubation with levansucrase at similar enzyme and substrate concentrations with the corresponding acceptor reaction (data not shown).

Our evidence highlights the capability of SacB to synthetize a complete series of neo-FOS from the corresponding precursor, i.e. 6-neo-nystose; however, during LMW levan synthesis only the tri- and tetra-saccharides accumulate, to be later consumed. This truncated polymerization may be related to the low acceptor specificity of neo-kestose, which also explains its accumulation among the primary trisaccharides. Our results also confirm the donor capacity of the neo-FOS, previously reported exclusively for neo-kestose^30^. The donor nature of the neo-FOS series oligosaccharides is not surprising, considering that all these products maintain the sucrose moiety in one end of their structure. Due to their lower specificity, the potential donor role of neo-kestose and 6-neo-nystose probably becomes important in the *sucrose depletion phase*. Blastose and blastotriose resulting from the corresponding fructosyl transfer or hydrolysis reactions may then be elongated to produce the secondary intermediates included in the LMW levan distribution.

### Network of transfer reactions during LMW levan synthesis

**Figure 6:**
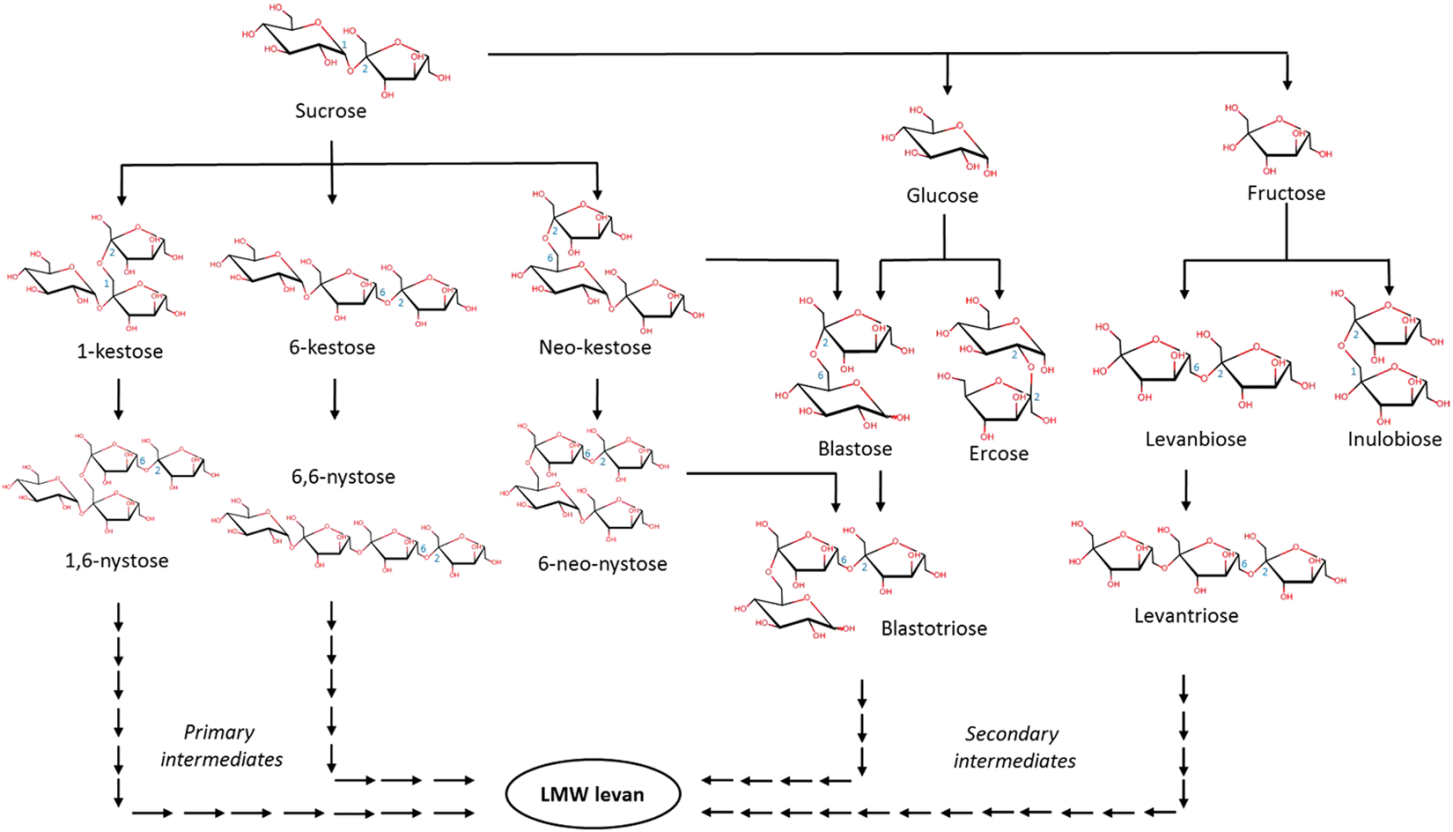
Diagram of the intermediates/products and fructosylation/hydrolysis reactions inherent to the *B. subtilis* levansucrase non-processive mechanism for low molecular weight (LMW) levan synthesis. The identified FOS series were classified as primary or secondary according to the reaction stage in which they are produced (see text).

LMW levan, obtained through a non processive synthesis reaction catalyzed by *B. subtilis* levansucrase, may be described as a set of FOS series that result from the elongation of different initiators synthesized from sucrose at different stages of the reaction mediated by the availability of different acceptors. The complex network of FOS series and initiators is schematized in Fig. 6. As described in detail in the previous sections, an *early phase* proceeds with the formation of 6-kestose, 1-kestose and neo-kestose as the first products of transfructosylation followed by the elongation of 6-kestose and 1-kestose to the corresponding levan series. Neo-kestose accumulates and is only consumed for the synthesis of 6-neo-nystose. 6K-FOS, 1K-FOS and two neo-FOS (neo-kestose and 6-neo-nystose) compose what we have defined as primary intermediates. In the *late phase*, when glucose and fructose reach an adequate concentration to become fructosyl acceptors, ercose, inulobiose, blastose, and levanbiose are produced, including the two latter as initiators of the blasto-FOS and oligolevan series respectively, both part of the secondary intermediate group of products. Finally, in the *sucrose depletion phase*, the primary intermediates neo-kestose and 6-neo-nystose are used as donors of fructosyl residues to produce blasto-FOS, increasing the concentration of these secondary intermediates. The final levan distribution is therefore a set of reducing and non-reducing fructan series whose structure depends on the acceptor used as initiator.

It is important to underline the variety of products synthesized by SacB in the *early phase* of the reaction. The presence of three products (6-kestose, 1-kestose and neo-kestose) reflects the low regioselectivity of levansucrase in the first fructosyl transfer to a sucrose molecule. The synthesis of these three kestoses has been reported in reactions catalyzed by both levansucrases and inulosucrases^24,27,31^, suggesting that this lack of regioselectivity when sucrose is the acceptor molecule may be a common characteristic among fructansucrases. The relaxed regiospecificity is consistent with the result of structure-function analysis that locate bond type specificity of fructansucrases in residues further away from the +2 substrate subsite^32^. Additionally, the broad acceptor specificity is also a common fructansucrase property outlined in the literature^33–35^. This relaxed specificity allows *B. subtilis* levansucrase to employ kestoses and even the products of sucrose hydrolysis (glucose and fructose) as acceptors, each according to its own specificity. Accordingly, SacB low regioselectivity explains the broad spectrum of compounds that comprise the low molecular weight levan distribution. In the same way, the multiplicity of intermediates and initiators resembles the reactions catalyzed by some glucansucrases, enzymes that produce glucose polymer from sucrose^36,37^, suggesting that these enzymes also have a relaxed acceptor specificity and low regioselectivity in the early transfer reactions.

In summary, this work contributes to a better understanding of the polymerization reaction of fructansucrases, clarifying the network of fructosyl transfer reactions involved in the non-processive biosynthesis of what has up to now been referred as low molecular weight levan, actually a distribution of various levan series. Further work to study the effect of oligosaccharide DPs in the reaction catalyzed by the *B. subtilis* levansucrase is in progress.

## Methods

### Standards and substrates

The following saccharides were used as standards: glucose, fructose, sucrose (Sigma Aldrich, USA); GFn DP3–10 inulin-type FOS OligoTech® (Elicityl, France); Oligofructose Orafti®P95 (Beneo, Germany), containing GFn and Fn DP 2–7 inulin-type FOS; 6-kestose and neokestose (kindly donated by Professor M. Iiusuka). The acceptor substrate1-kestose was purchased from Wako Pure Chemical Industries (Japan); levanbiose was obtained as recently reported^38^; and blastose, 6-kestose, neo-kestose and 6-neo-nystose were isolated (5–10 mg) from SacB reactions and purified by size exclusion chromatography (SEC) followed by phase-reverse chromatography (rpHPLC). SEC fractionation was performed in a Bio-Gel P2 Extra Fine column (2.5 × 100 cm, Bio-Rad) using MQ water as eluent at 0.2 mL/min. rpHPLC was carried out in a Waters 1525 HPLC system equipped with a Spherisorb S5 ODS2 Semi-Prep column (20 × 250 mm, Waters), employing MQ water as mobile phase at 7 mL/min.

### Recombinant expression and purification of SacB

*Escherichia coli* BL21 (DE3) harboring pET22b + *sacB*^39^ was grown to mid-exponential phase at 37 °C with aeration until A_600_ reached 0.6. Expression of SacB was induced by adding isopropylthio-β-D-galactoside (IPTG) at a concentration of 0.2 mM and then incubated for 8 h at 18 °C. The recombinant enzyme was purified from the cell soluble fraction using cation exchange chromatography according to a previously reported protocol^19^. Enzyme purity was corroborated by SDS-PAGE and protein content quantified employing the Bradford reagent (Bio-Rad) with albumin as standard.

### SacB and acceptor reactions

We carried out the levansucrase reactions under enzyme and sucrose concentrations that favors the exclusive synthesis of LMW levan according to our previous work^19^, i.e. 0.5 µM (27.5 µg mL^−1^) enzyme and 292 mM (100 g L^−1^) sucrose, in a medium containing 50 mM acetate buffer (pH 6) and 1 mM CaCl_2_. We verified the exclusive synthesis of LMW levan through gel permeation chromatography analysis as previously reported^19^. The acceptor reactions were carried out adding 5 mM of 1-kestose, 6-kestose, or 6-neo-nystose to the reaction mixture. In the case of blastose, levanbiose, and neo-kestose acceptor reactions, we increased the acceptor concentration to 20 mM to improve acceptor reaction efficiency. The reaction mixtures were allowed to proceed at 25 °C with aliquots removed at different time intervals. Samples were immediately frozen, and later heated in boiling water during 10 min for enzyme denaturalization and stored at −20 °C until analysis. Additionally, the acceptors were incubated exclusively with SacB to evaluate both, their donor capacity and their eventual hydrolysis. All experiments were performed by duplicate.

### Oligosaccharide analysis and quantification

We obtained the oligosaccharide profiles by HPAE-PAD analysis in a Dionex DX-500 IC system equipped with GP50 pump, ED50 electrochemical detector, U3000 autosampler and a CarboPac PA200 column (3 × 250 mm, Dionex) maintained at 30 °C. Product elution was carried out applying a sodium acetate gradient with 100 mM NaOH at 0.5 mL min^−1^ as follows: 5–100 mM sodium acetate in 25 min, 100–400 mM in 60 min, and 10 min for initial condition reequilibration (5 mM sodium acetate). The initial identification of some oligosaccharides was achieved by comparison with standards and, in the case of blastose and levanbiose, according to HPAE-PAD analysis previously reported^27,38^. The oligosaccharides 1-kestose, 6-kestose, neo-kestose, levanbiose and blastose were also employed in the generation of standard curves for quantitative analysis through HPAE-PAD.

Simple sugars (fructose, glucose, and sucrose) were analyzed in a Waters 1525 HPLC system equipped with Waters 717 plus autosampler and Waters 2414 refractive index detector. We employed a Prevail Carbohydrate ES column (4.6 × 250 mm) kept at 30 °C and acetonitrile-water (75:25, v/v) as mobile phase at 1 mL/min. All measurements were performed by duplicate.

### NMR analysis

NMR spectra were acquired on a Varian Unity NMR spectrometer operating at 700 MHz for ^1^H and 125 MHz for ^13^C nuclei. Chemical shifts are listed in parts per million (ppm), referenced to D_2_O and were assigned on the basis of ^1^H–^1^H gCOSY, gTOCSY, gHSQC, and gHMBC spectral analysis as required.

## Electronic supplementary material

Supplementary Figures and Information
