# Supplementary material for: Understanding the transfer reaction network behind the non-processive synthesis of low molecular weight levan catalyzed by Bacillus subtilis levansucrase
Source: Sci Rep. 2018 Oct 9;8:15035. doi: 10.1038/s41598-018-32872-7 (PMC6177408; doi:10.1038/s41598-018-32872-7)

Understanding the transfer reaction network behind the non-processive synthesis of low molecular weight levan catalyzed by *Bacillus subtilis* levansucrase

Enrique Raga-Carbajal[1], Agustín López-Munguía[1], Laura Alvarez[2] and Clarita Olvera[1]*

[1]Departamento de Ingeniería Celular y Biocatálisis, Instituto de Biotecnología, UNAM. Av. Universidad #2001, Col. Chamilpa, C. P. 62210, Cuernavaca, Morelos, México.

[2]Centro de Investigaciones Químicas-IICBA, Universidad Autónoma del Estado de Morelos. Av. Universidad #1001, Col. Chamilpa, C.P. 62210, Cuernavaca, Morelos, México

*Correspondence and requests for materials should be addressed to CO (clarita@ibt.unam.mx).

Supplementary Figures

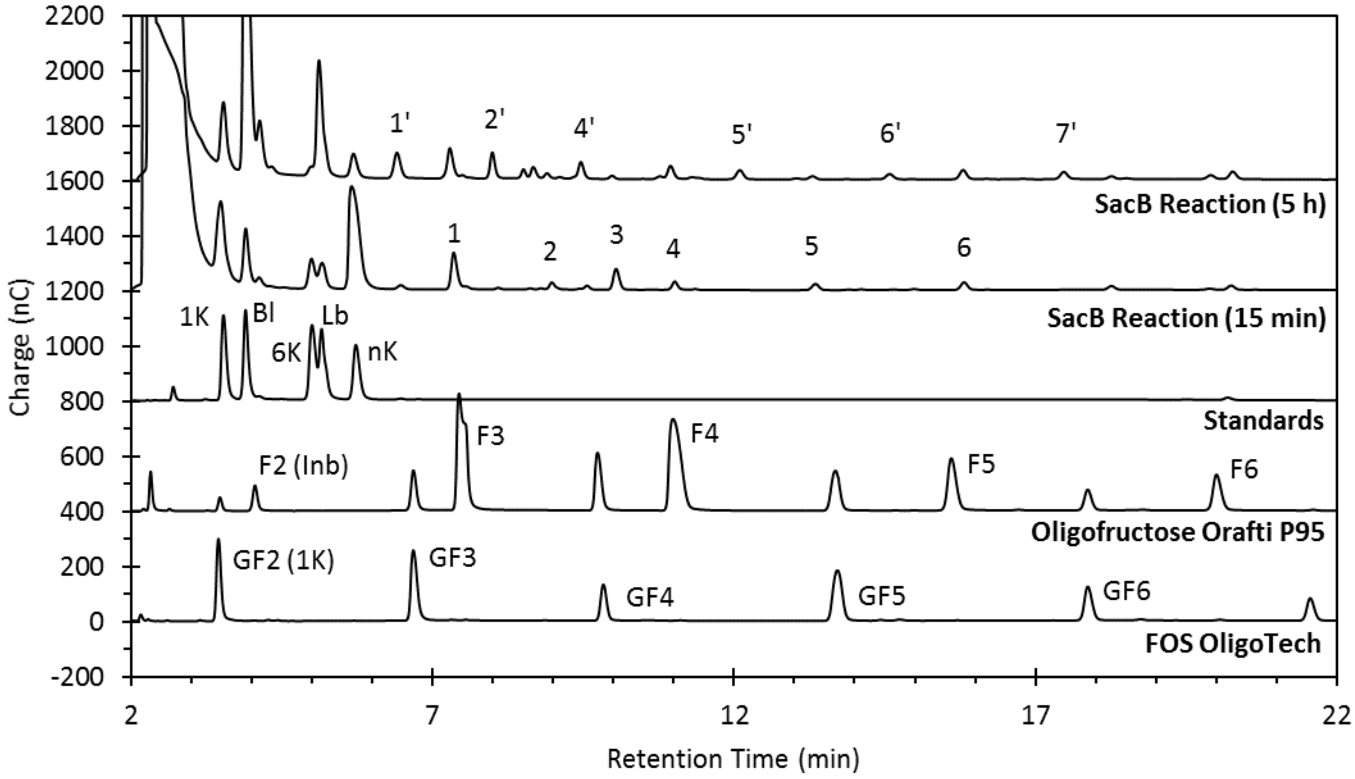

Figure S1. Comparison of SacB reaction profile with standards and commercial inulin-type FOS. 1K: 1-kestose; Bl: blastose; 6K: 6-kestose; Lb: levanbiose; nK: neo-kestose; F2 (Inb): inulobiose; Fn: inulin-type FOS containing only fructose units; GFn: inulin-type FOS containing initial glucose unit; 1-6: primary intermediates; 1'-7': secondary intermediates.

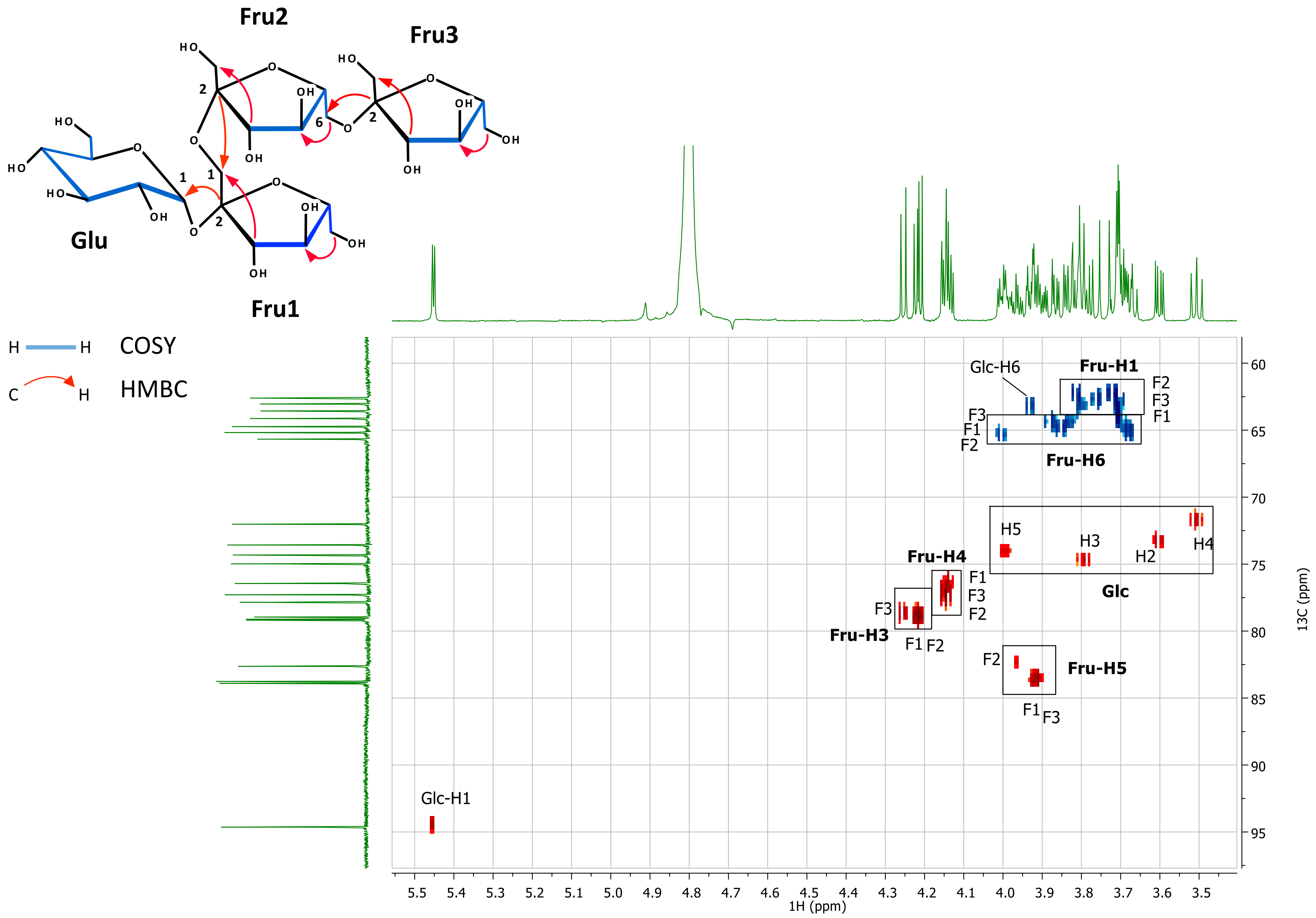

Figure S2. HSQC spectrum of 1,6-Nystose (Peak 1). The signals are labeled and the key points for identification are schematized.

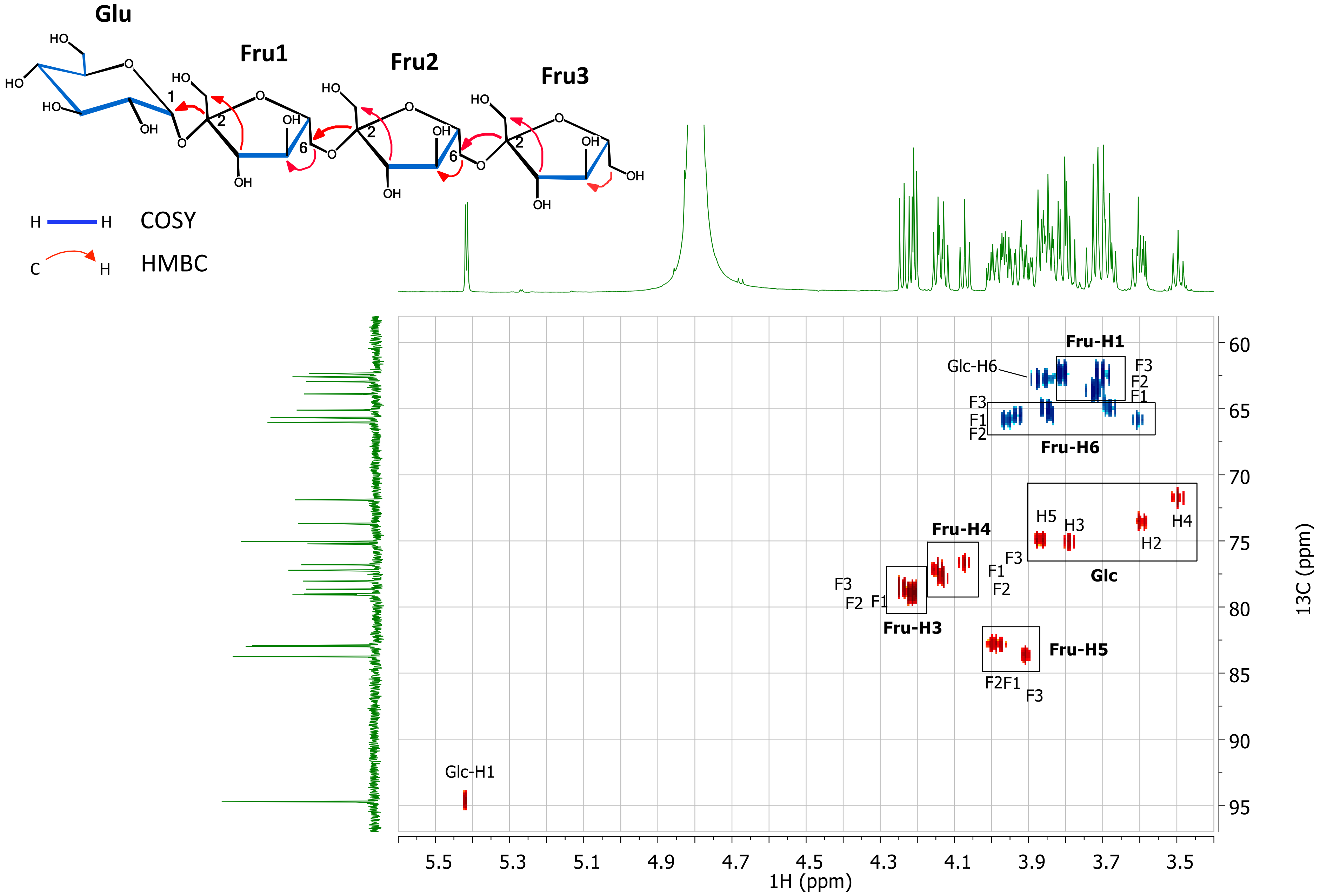

Figure S3. HSQC spectrum of 6,6-Nystose (Peak 2). The signals are labeled and the key points for identification are schematized.

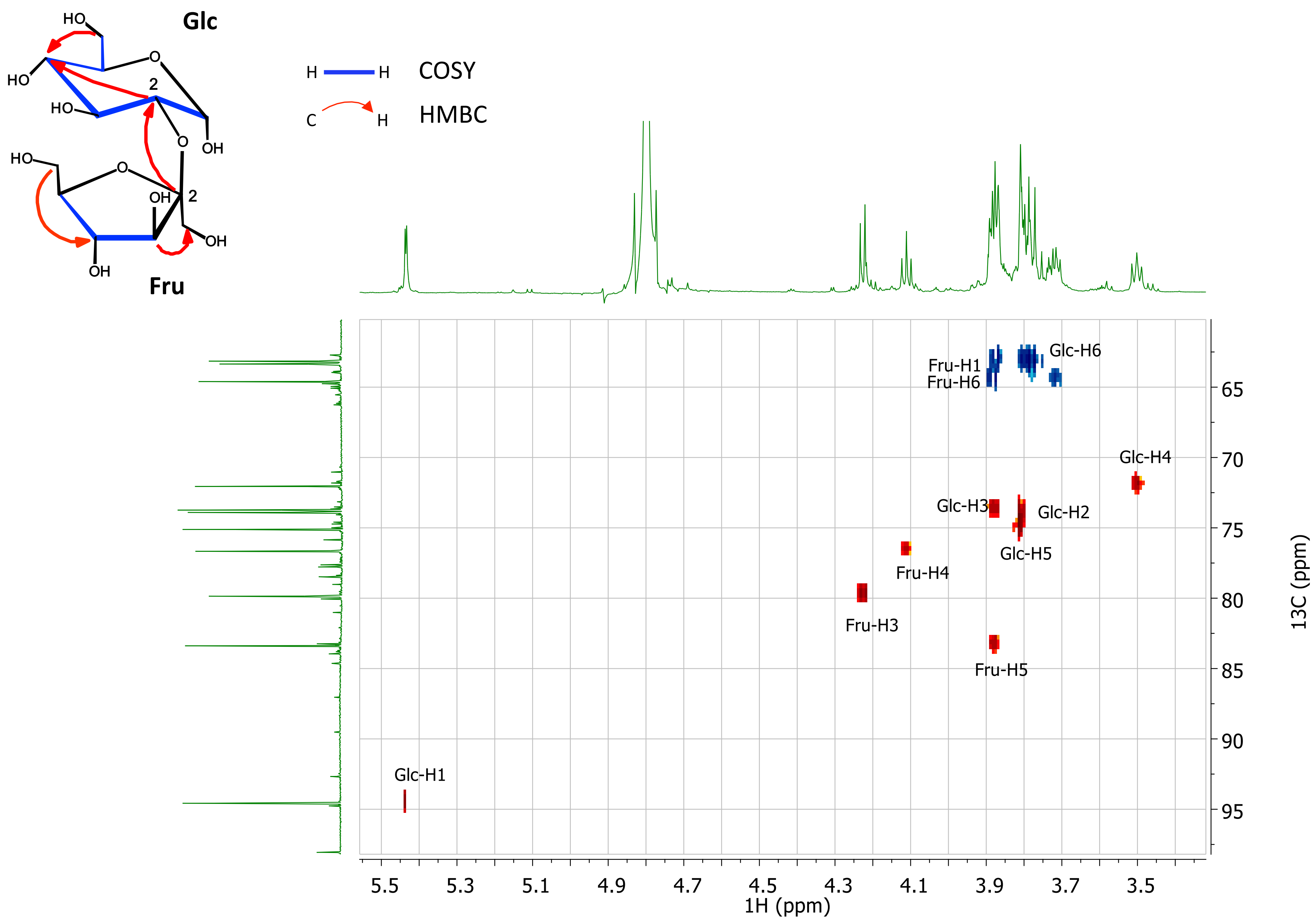

Figure S4. HSQC spectrum of β2-2 Sucrose isomer (Peak 1'). The signals are labeled and the key points for identification are schematized.

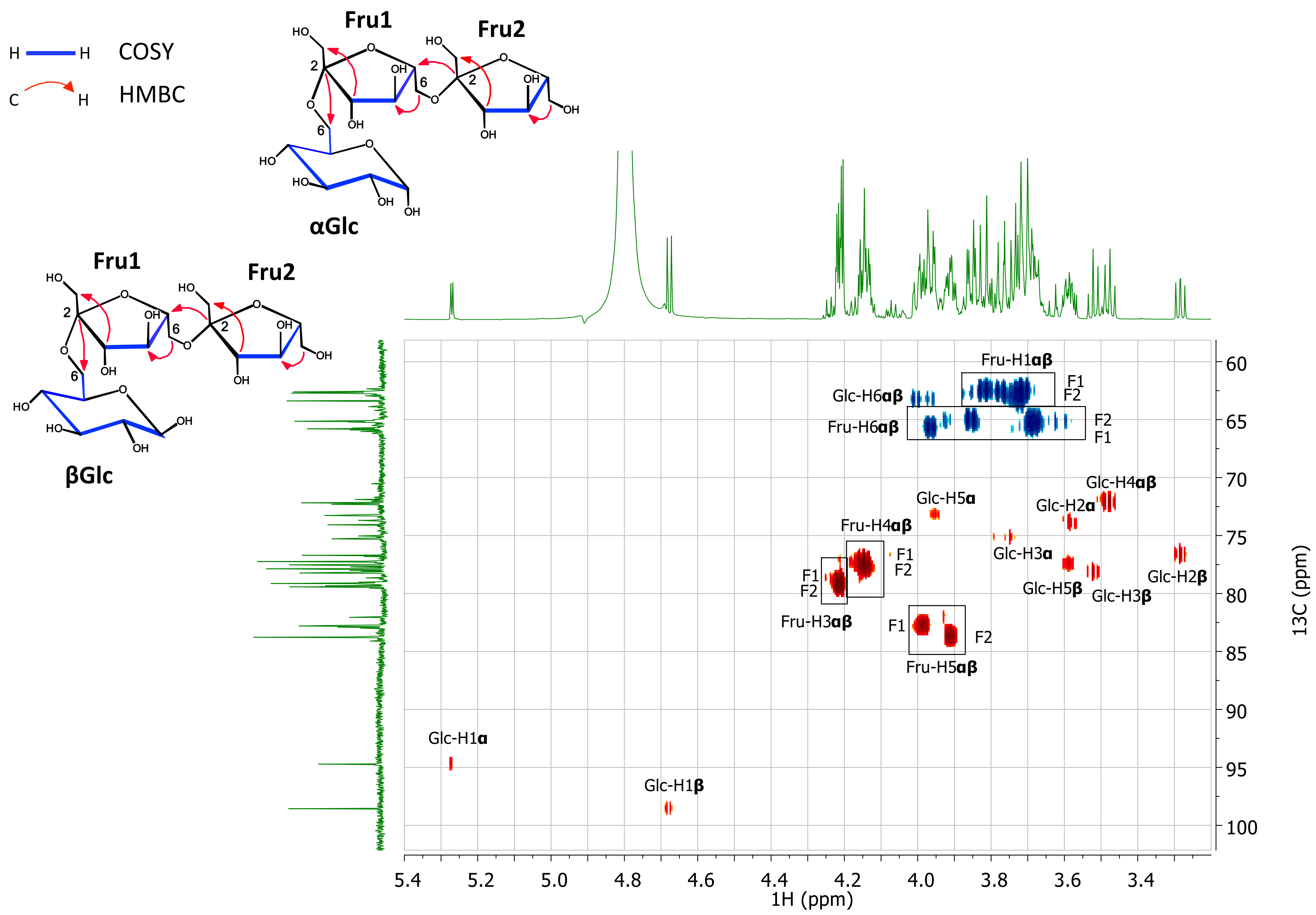

Figure S5. HSQC spectrum of α/β-Blastotriose (Peak 2’). The signals are labeled and the key points for identification are schematized.

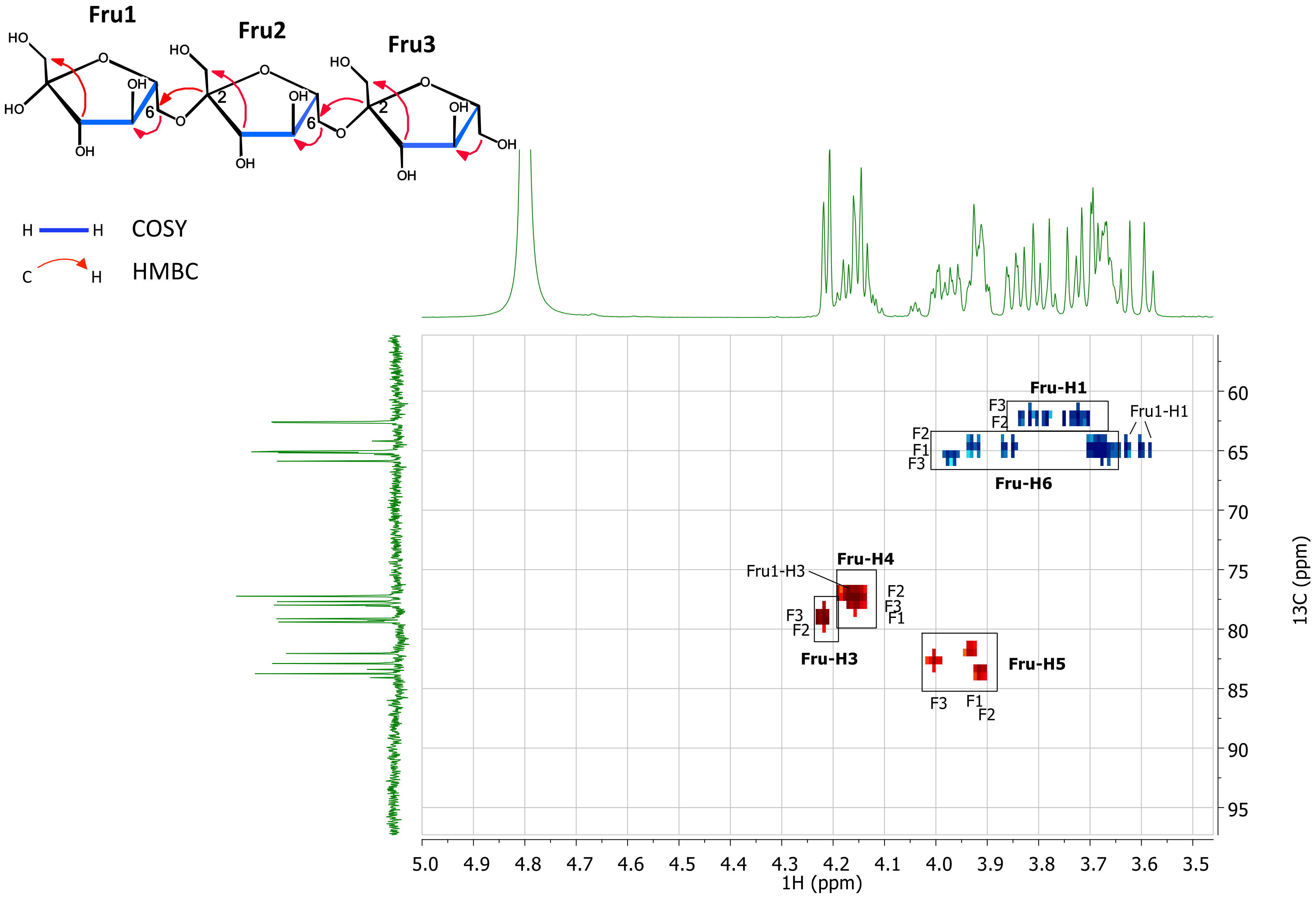

Figure S6. HSQC spectrum of Levantriose (Peak 4'). The signals are labeled and the key points for identification are schematized.

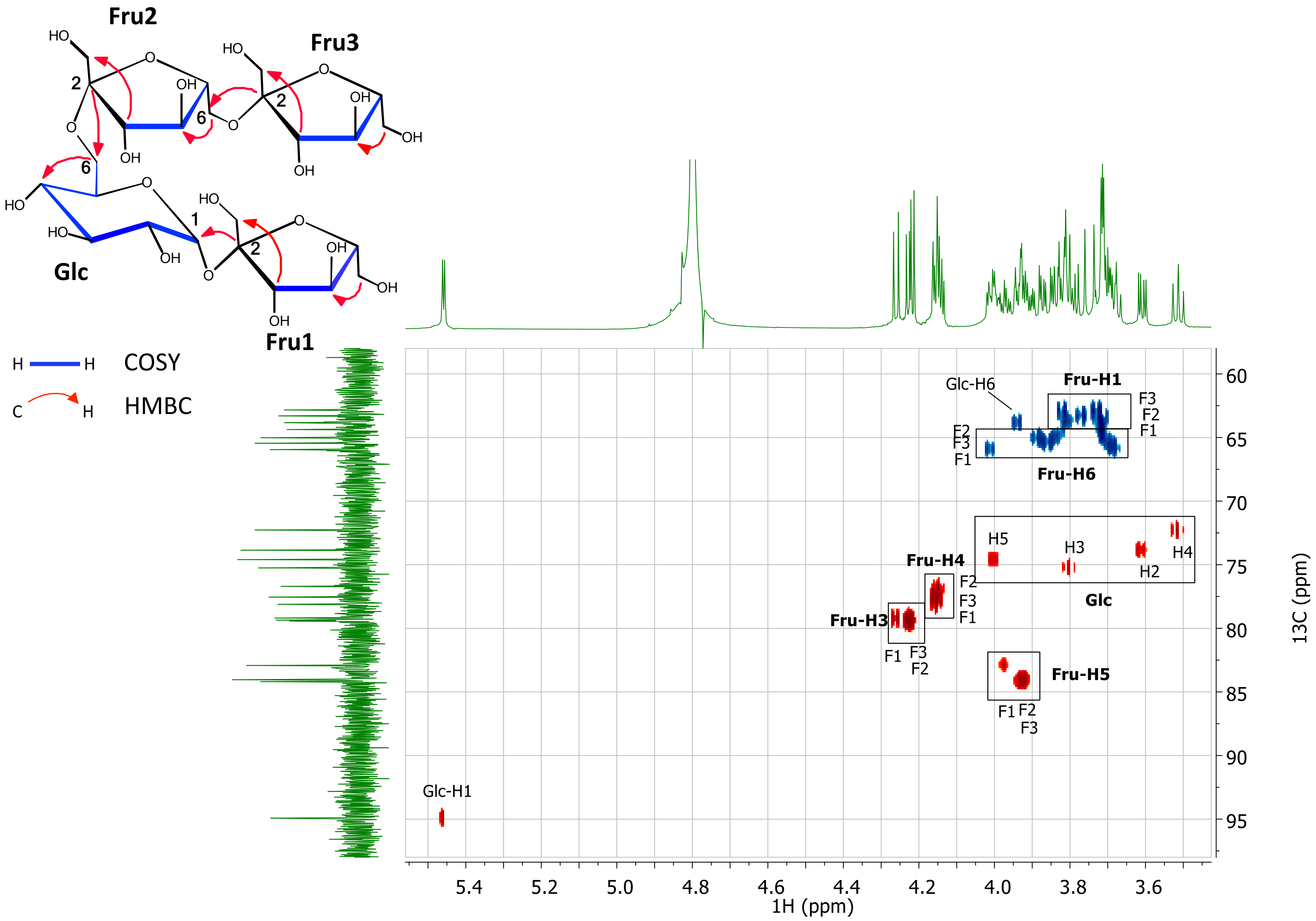

Figure S7. HSQC spectrum of 6-neo-Nystose (Peak 3). The signals are labeled and the key points for identification are schematized.

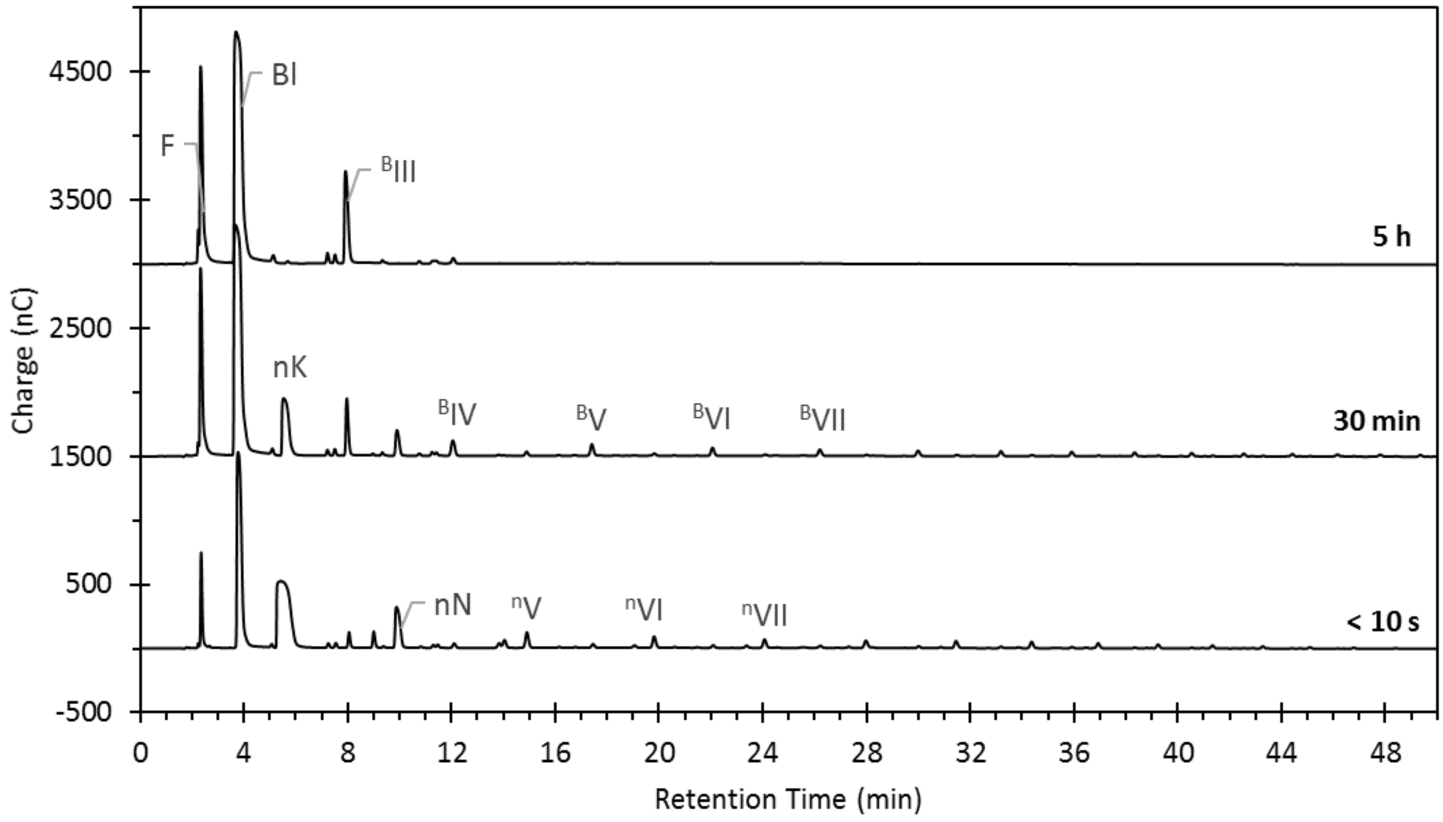

Figure S8. Reaction of SacB with neo-kestose (nK) as sole substrate analyzed by HPAE-PAD. At the beginning of the reaction, neo-kestose is able to act as both donor and acceptor substrates yielding blastose (Bl) as leaving group and neoFOS (^{n}V-^{n}VII), including 6-neo-nystose (nN) as first product of transfructosylation. Afterwards, the neoFOS are consumed whereas blasto-FOS (^{B}III-^{B}VII) emerge concurrently. At the end of the reaction three products remain: blastose, blastotriose (^{B}III) and fructose (F), the last as product of continuous hydrolysis. Reaction conditions: SacB 0.5 µM, neo-kestose 25 mM, pH 6, 25°C.

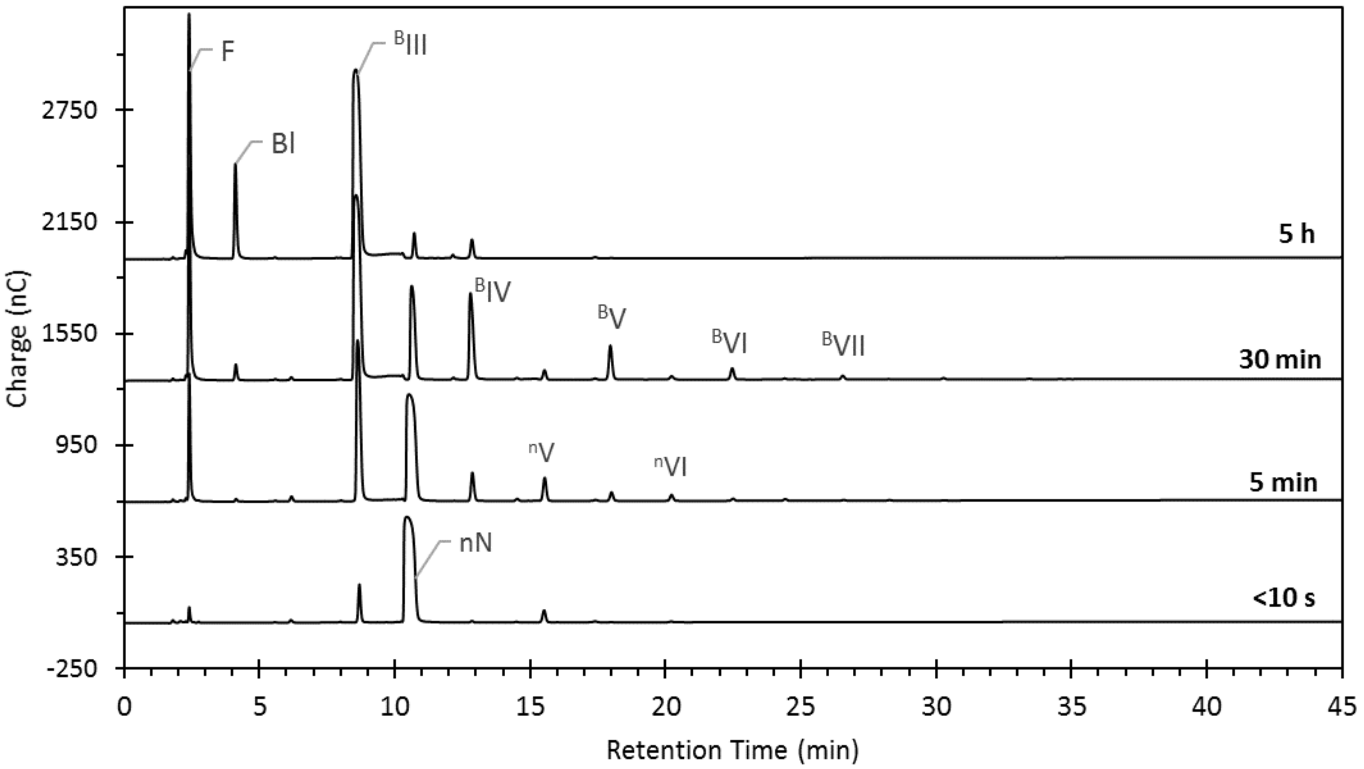

Figure S9. Reaction of SacB with 6-neo-nystose (nN) as sole substrate followed by HPAE-PAD analysis. The tetrasaccharide 6-neo-nystose acts as donor and acceptor substrates yielding blastotriose (^{B}III) as leaving group. In a similar way to the neo-kestose reaction, neoFOS (^{n}V-^{n}VI) are firstly produced and consumed with the concurrent formation of blasto-FOS (^{B}III-^{B}VII). The three mainly products at the end of the reaction are: blastose (Bl), blastotriose (^{B}III) and fructose (F). Reaction conditions: SacB 0.5 µM, 6-neo-nystose 5 mM, pH 6, 25°C.

Understanding the transfer reaction network behind the non-processive synthesis of low molecular weight levan catalyzed by *Bacillus subtilis* levansucrase

Enrique Raga-Carbajal[1], Agustín López-Munguía[1], Laura Alvarez[2] and Clarita Olvera[1]*

[1]Departamento de Ingeniería Celular y Biocatálisis, Instituto de Biotecnología, UNAM. Av. Universidad #2001, Col. Chamilpa, C. P. 62210, Cuernavaca, Morelos, México.

[2]Centro de Investigaciones Químicas-IICBA, Universidad Autónoma del Estado de Morelos. Av. Universidad #1001, Col. Chamilpa, C.P. 62210, Cuernavaca, Morelos, México

*Correspondence and requests for materials should be addressed to CO (clarita@ibt.unam.mx).

Appendix A. NMR Spectra

Table A1. NMR data for the identification of SacB products.

	6,6-Nystose		1,6-Nystose		6-neo-Nystose	
C-atom	δC	δH	δC	δH	δC	δH
C-1	94.74	5.42, d (3.5)	94.63	5.45, d (4.2)	94.92	5.46, d (3.83)
C-2	73.69	3.61-3.58, m	73.57	3.60, dd (9.8, 3.5)	73.85	3.61, dd 3.89,10.00)
C-3	75.23	3.77-3.80, m	74.98	3.79, dd (9.1)	75.25	3.79, m
C-4	71.89	3.49, t (9.1)	72.01	3.50, dd (9.8)	72.27	3.51, t (9.64)
C-5	75.03	3.85-3.87, m	74.31	3.99, m	74.60	4.00, m
C-6	62.94	3.87-3.48, m	63.56	3.93, dd (11.2, 2.1)	63.83	3.93, m
				3.70, m		3.81, m
C-1’	63.88	3.72 d (9.0)	64.13	3.70, m	64.37	3.71, m
				3.80, m		
C-2’	106.39	---	106.62	---	106.62	---
C-3’	79.02	4.20, d (8.4)	79.10	4.22, d (8.4)	79.19	4.26, d (8.80)
C-4’	77.22	4.14, t (8.4)	76.44	4.14, t (8.6)	78.11	4.16, t (8.64)
C-5’	82.98	3.98-4.01, m	83.75	3.91 ddd (4.2, 3.5)	82.92	3.97, m
C-6’	65.66	3.95, dd (10.5, 3.5)	65.17	3.86, d (11.2)	65.94	4.01, m
		3.60-3.59, m		3.67, m		3.68, m
C-1”	62.34	3.68, m	62.60	3.81, dd (11.2, 2.8)	63.29	3.77, m
		3.84, m		3.72, m		
C-2”	106.39	---	106.33	---	106.91	---
C-3”	79.08	4.21, d (8.4)	79.18	4.21, d (8.4)	79.41	4.21, d (8.46)
C-4”	78.04	4.12, t (8.4)	77.85	4.14, t (8.4)	76.70	4.14, t (8.12)
C-5”	82.91	3.98-4.01, m	82.63	3.96, ddd (7.7, 3.5)	84.18	3.96, m
C-6”	66.03	3.95, dd (10.5, 3.5)	65.67	4.00, m	65.01	3.89, m
		3.60-3.59, m		3.67, m		3.84, m
C-1”’	62.58	3.79 d (9.1)	63.04	3.76, d (11.2)	62.83	3.82, m
		3.70 d (10.5)		3.72, d (11.2)		3.72, m
C-2”’	106.76	---	106.41	---	105.70	---
C-3‘”	78.65	4.24, d (8.5)	78.94	4.25, d (8.4)	79.35	4.22, d (8.49)
C-4”’	76.80	4.07, t (8.4)	77.28	4.14, t (8.4)	77.54	4.15, t (8.12)
C-5”’	83.75	3.89-3.91, m	83.90	3.91 ddd (4.2, 3.5)	84.04	3.92, m
C-6”’	65.10	3.85 dd (11.9, 3.5)	64.73	3.88, m	65.44	3.86, m
		3.68-3.66, m		3.82, m		

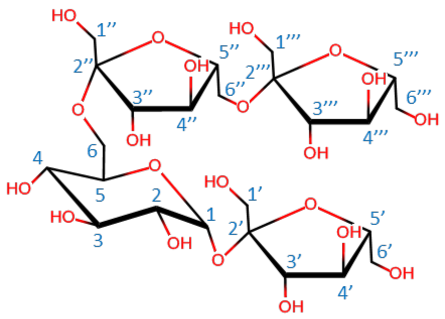

6-neo-nystose
(Peak 3)

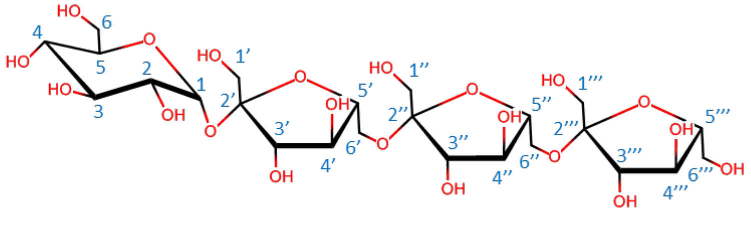

6,6-nystose
(Peak 2)

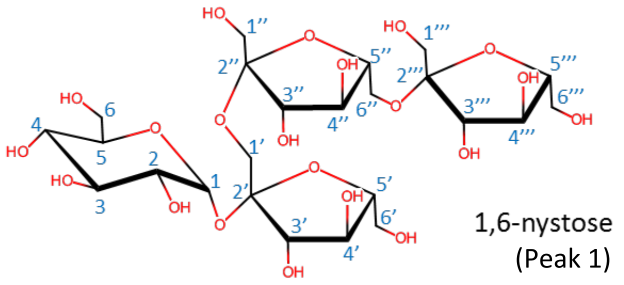

1,6-nystose
(Peak 1)

Table A1 (continuation). NMR data for the identification of SacB products.

C-atom	Ercose δC	Ercose δH	Levantriose δC	Levantriose δH	α-Blastotriose δC	α-Blastotriose δH	β-Blastotriose δC	β-Blastotriose δH
C-1	94.57	5.44, d (2.9)	65.17	3.58, d (12.6)	94.72	5.27, d (3.76)	98.56	4.67, d (7.7)
				3.63, d (12.6)				
C-2	73.90	3.80, m	104.39	---	74.07	3.57, m	76.70	3.28 dd (9.1, 7.7)
C-3	73.73	3.87, dd (10.5, 9.8)	77.24	4.16, d (7)	75.23	3.73, t (10.5, 10.5)	78.23	3.52 dd (9.1, 9.1)
C-4	72.05	3.49, dd (9.8)	77.98	4.18, dd (8.4, 7)	72.30	3.47, t (10.5)	72.17	3.49 dd (9.1, 9.1)
								3.86
C-5	75.11	3.81, m	82.05	3.90, m	73.27	3.95, ddd (2.8, 7.7)	77.52	3.58, m
C-6	63.15	3.75, m	65.10	3.65, m	63.38	3.99, m	63.38	3.99, m
		3.73, m				3.85, dd (2.8, 12.2)		3.85, dd (2.8, 12.2)
C-1’	63.36	3.79, m	62.64	3.82, d (11.9)	62.71	3.72, d (11.9)	62.71	3.72, d (11.9)
		3.87, m		3.73, d (11.9)		3.85, d (12.2)		3.85, d (12.2)
C-2’	106.13	---	106.58	---	106.56	---	106.56	---
C-3’	79.86	4.22, d (8.7)	79.40	4.20, d (7.7)	79.10	4.21, d (8.5)	79.10	4.21, d (8.5)
C-4’	76.67	4.11, t (8.5)	77.23	4.14, dd (7.7)	77.87	4.14, m	77.87	4.14, m
C-5’	83.38	3.87, m	83.75	3.91, m	82.80	3.99, m	82.80	3.99, m
C-6’	64.60	3.71, m	65.04	3.86, dd (2.8, 11.9)	65.79	3.99, m	65.79	3.99, m
		3.88, m		3.92, m				
C-1”			62.57	3.79 d (12.6)	62.61	3.82, d (12.6)	62.61	3.82, d (12.6)
				3.7 d (12.6)		3.77, d (12.6)		3.77, d (12.6)
C-2”			106.38	---	106.39	---	106.39	---
C-3”			79.12	4.21, d (7)	79.42	4.20, d (8.5)	79.42	4.20, d (8.5)
C-4”			77.68	4.13, dd (7.7)	77.24	4.14, m	77.24	4.14, m
C-5”			82.89	4.0, dd (8.4, 2.8)	82.90	3.97, m	82.90	3.97, m
C-6”			65.88	3.96, dd (2.8, 10.5)	65.15	3.85, dd (3.5, 11.9)	65.13	3.70, dd (2.8, 12.6)
				3.69, m		3.77, dd (2.8, 12.6)		3.58, m 3.85, 3.69

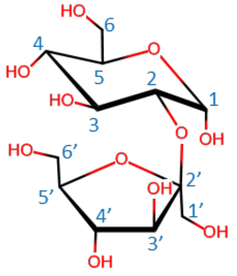

Ercose
(Peak 1’)

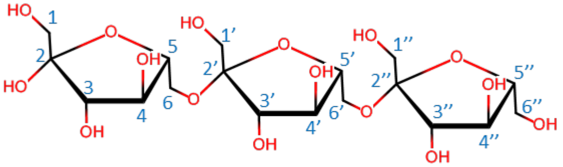

Levantriose
(Peak 4’)

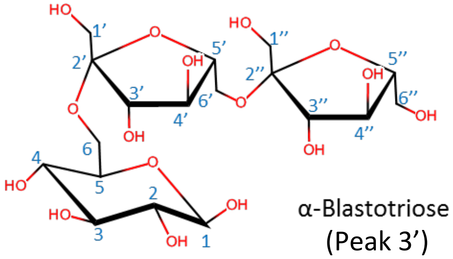

α-Blastotriose
(Peak 3’)

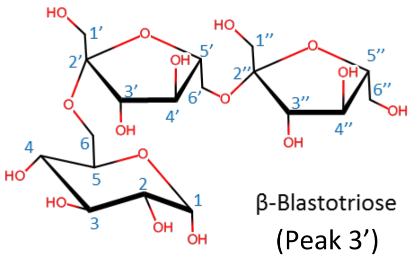

β-Blastotriose
(Peak 3’)

1H –1,6-Nystose (Peak 1)

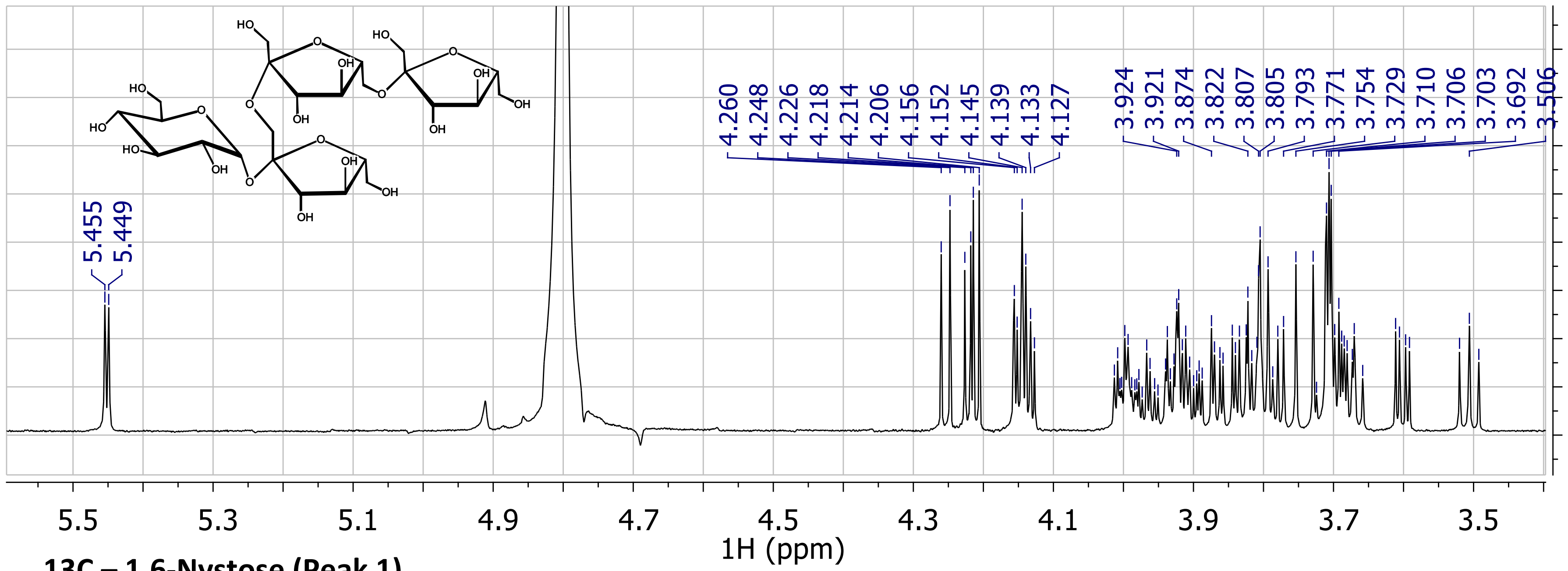

13C – 1,6-Nystose (Peak 1)

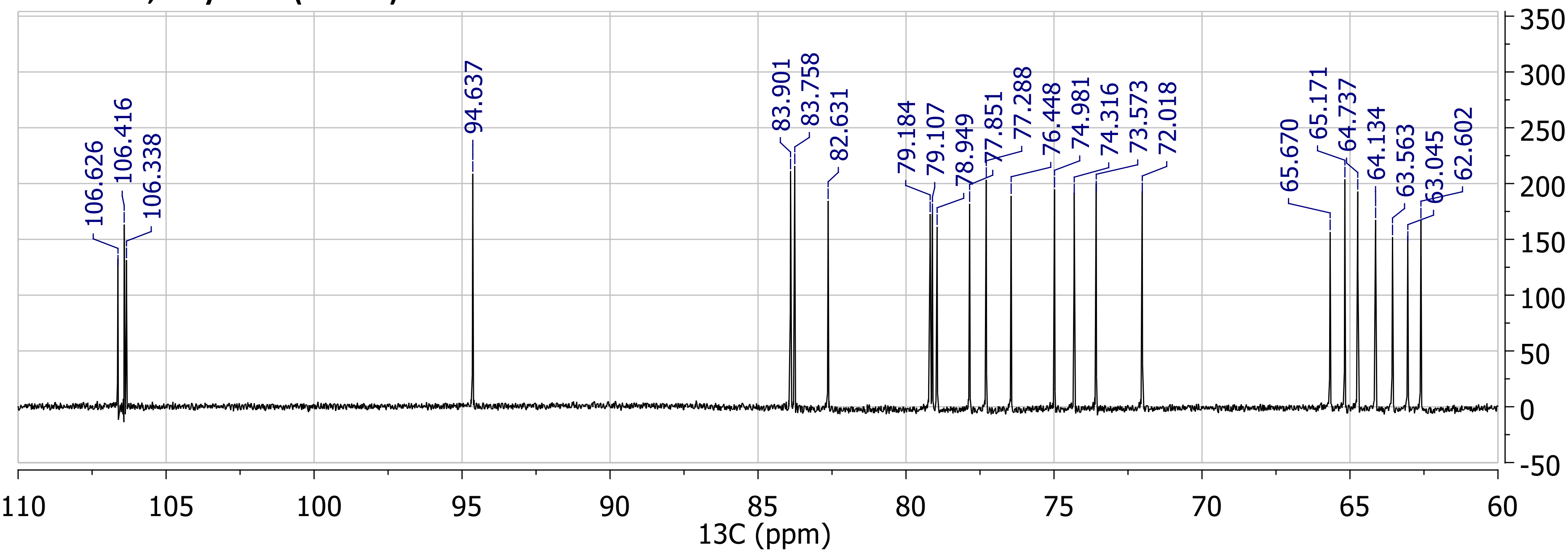

HSQC – 1,6-Nystose (Peak 1)

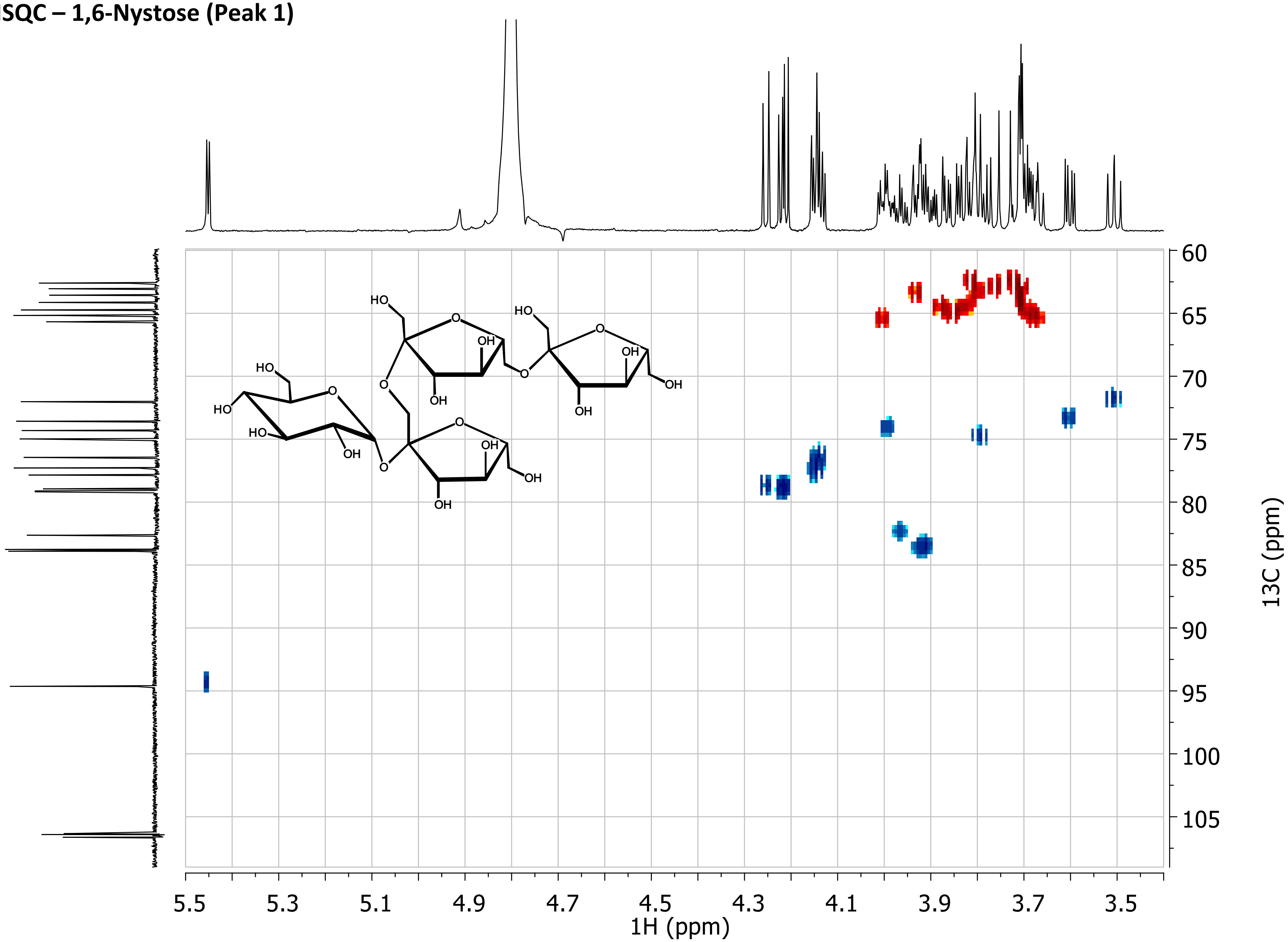

COSY – 1,6-Nystose (Peak 1)

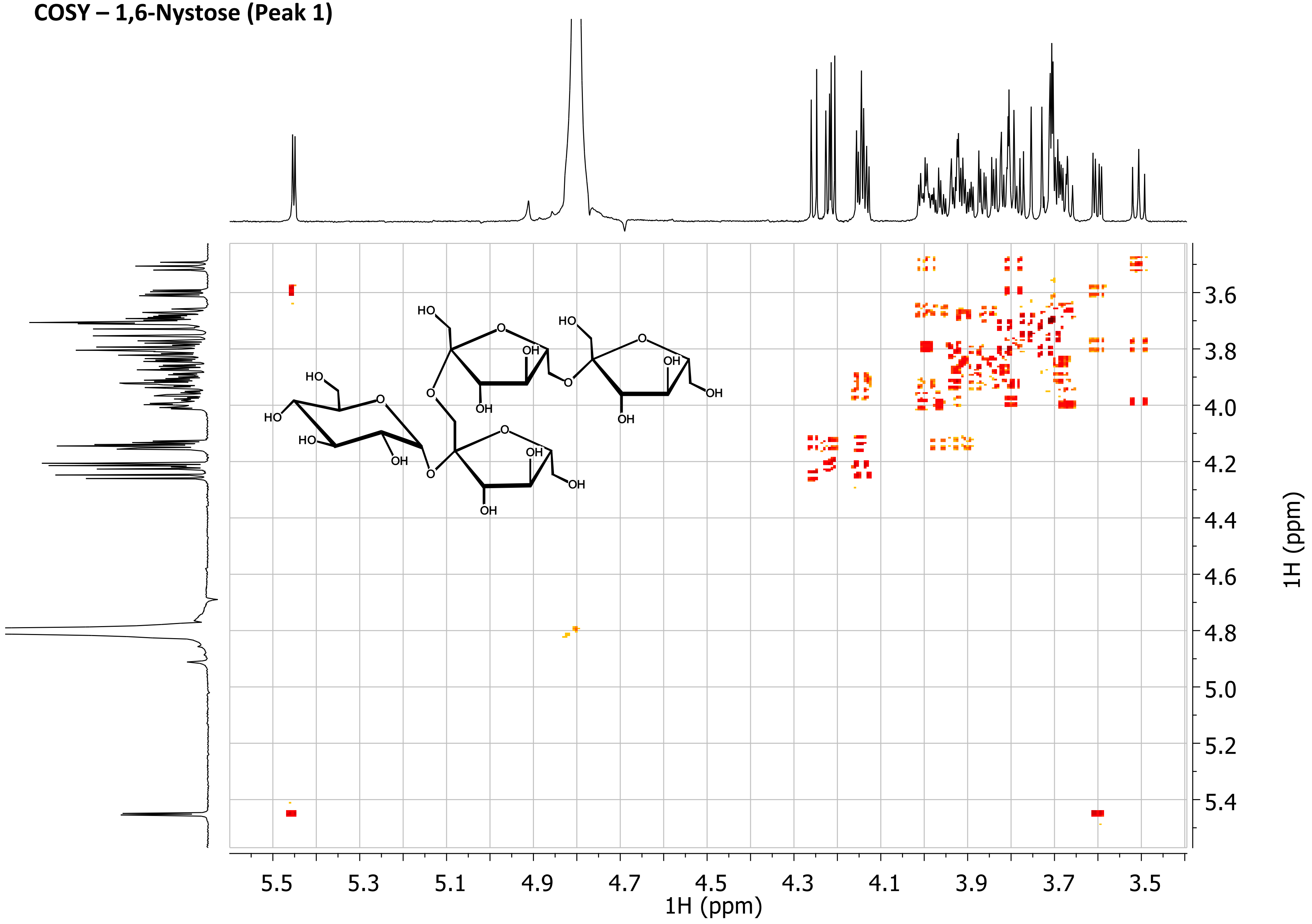

HMBC – 1,6-Nystose (Peak 1)

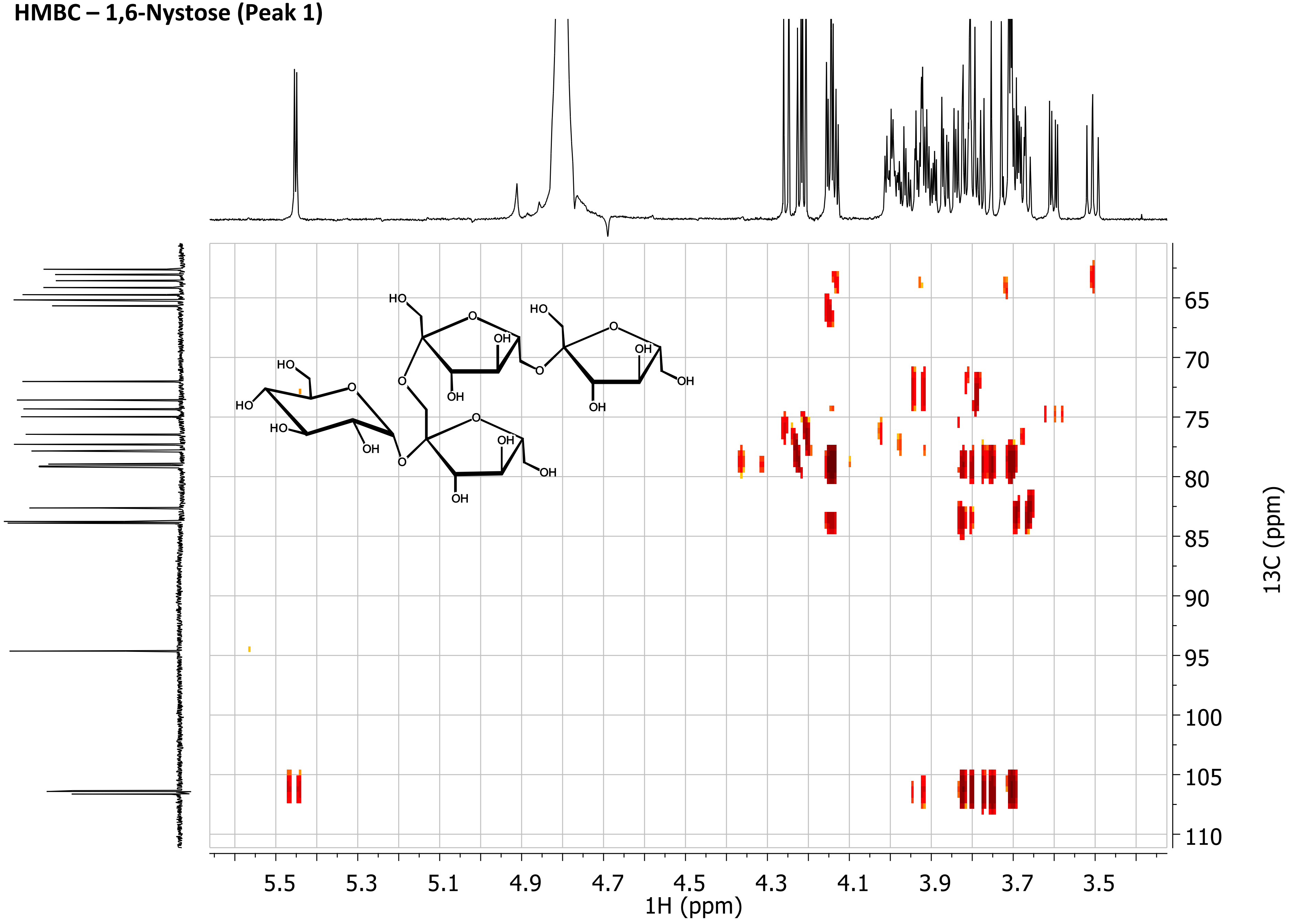

1H – 6,6-Nystose (Peak 2)

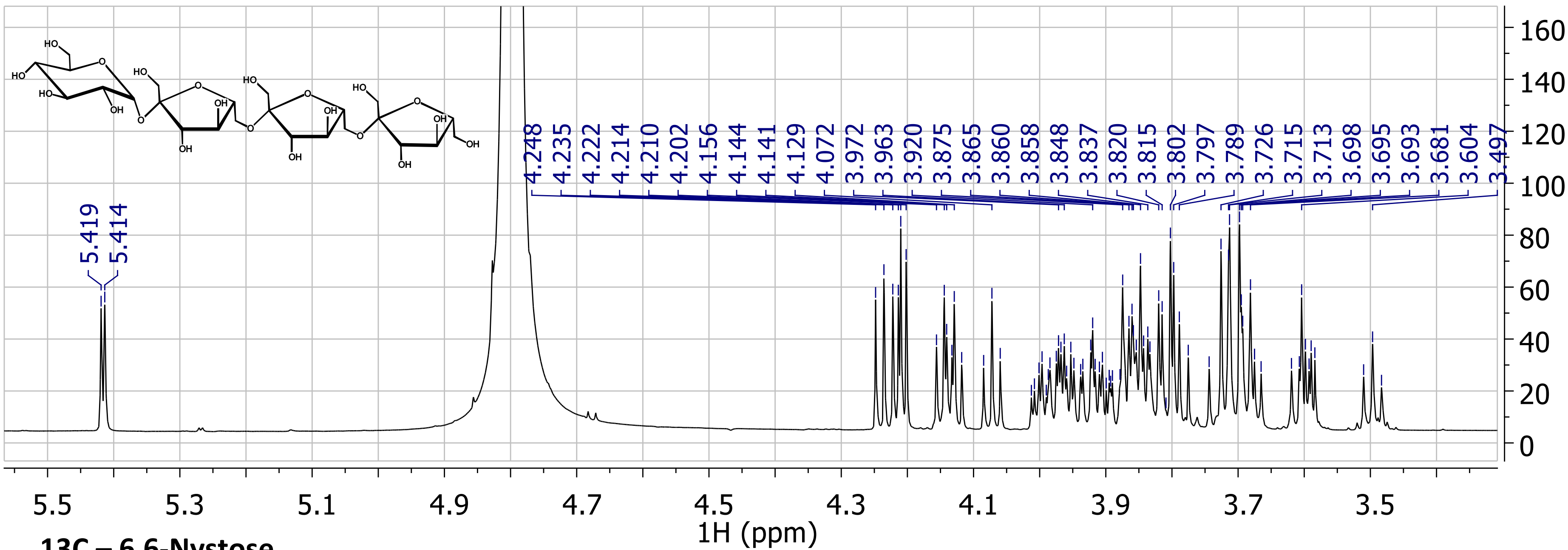

13C – 6,6-Nystose

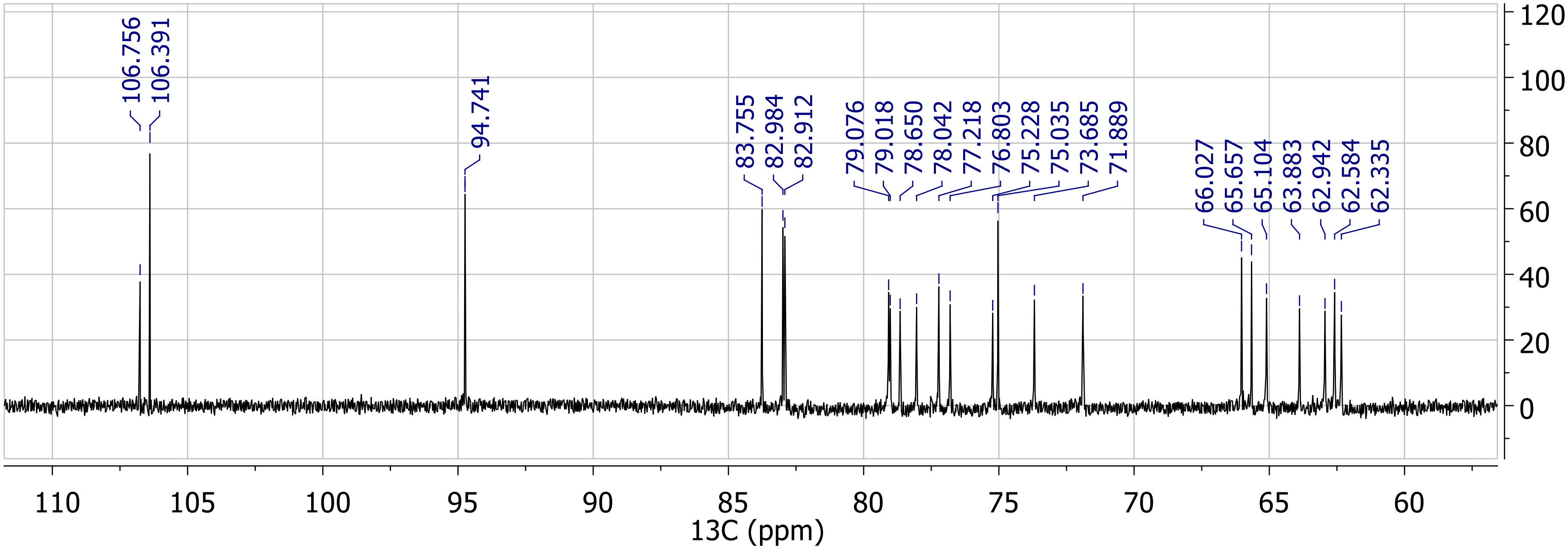

HSQC – 6,6-Nystose (Peak 2)

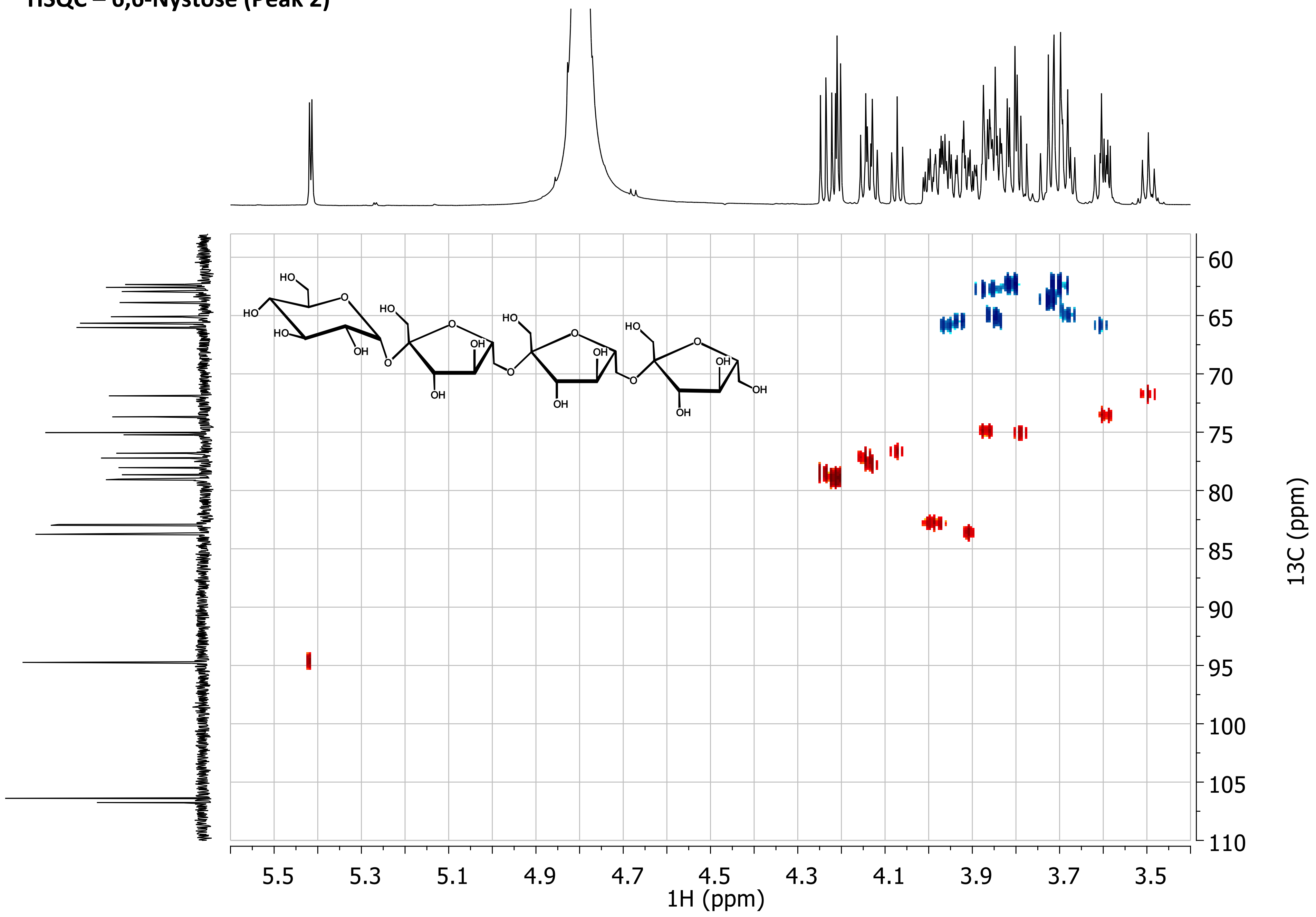

COSY – 6,6-Nystose (Peak 2)

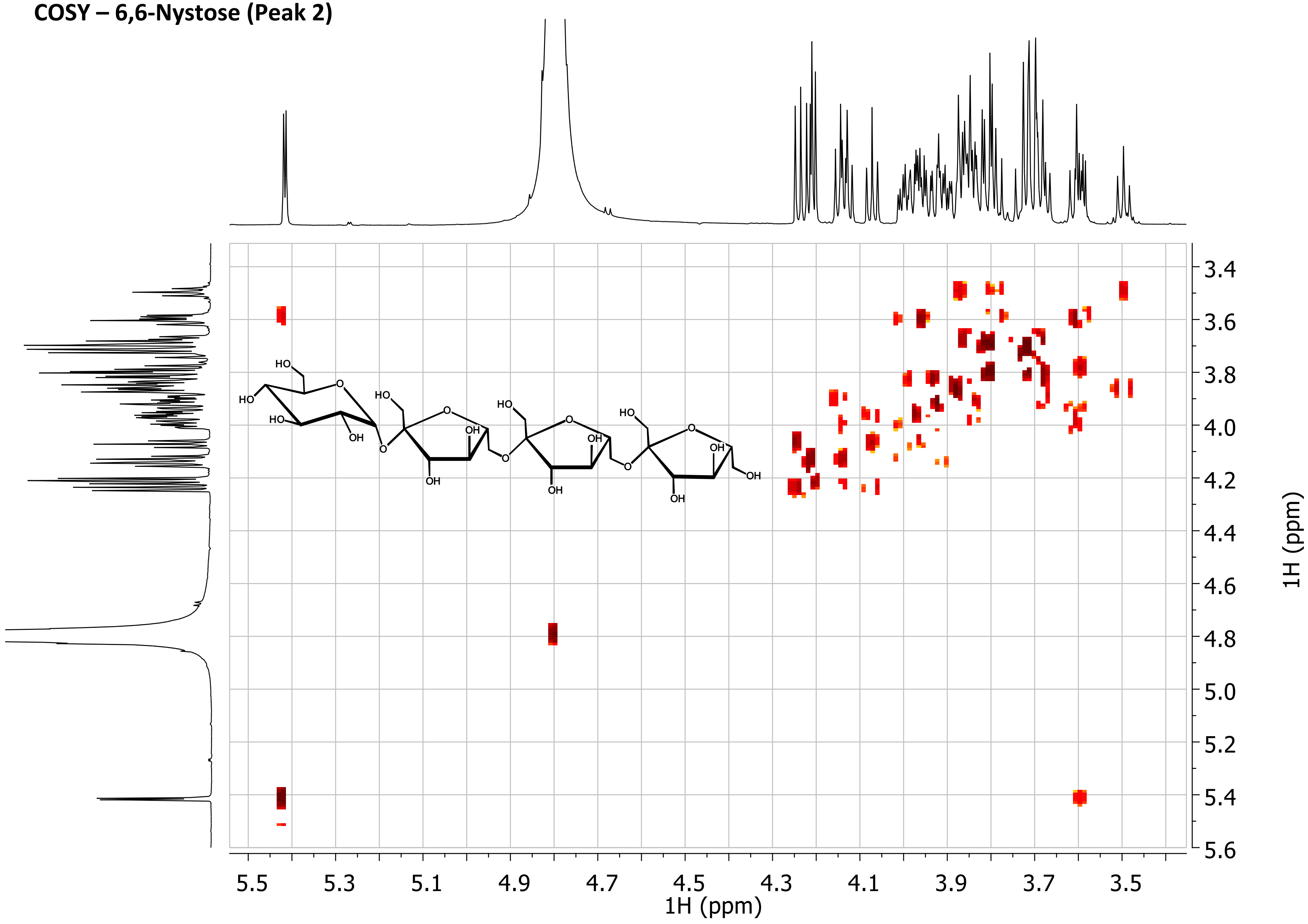

HMBC – 6,6-Nystose (Peak 2)

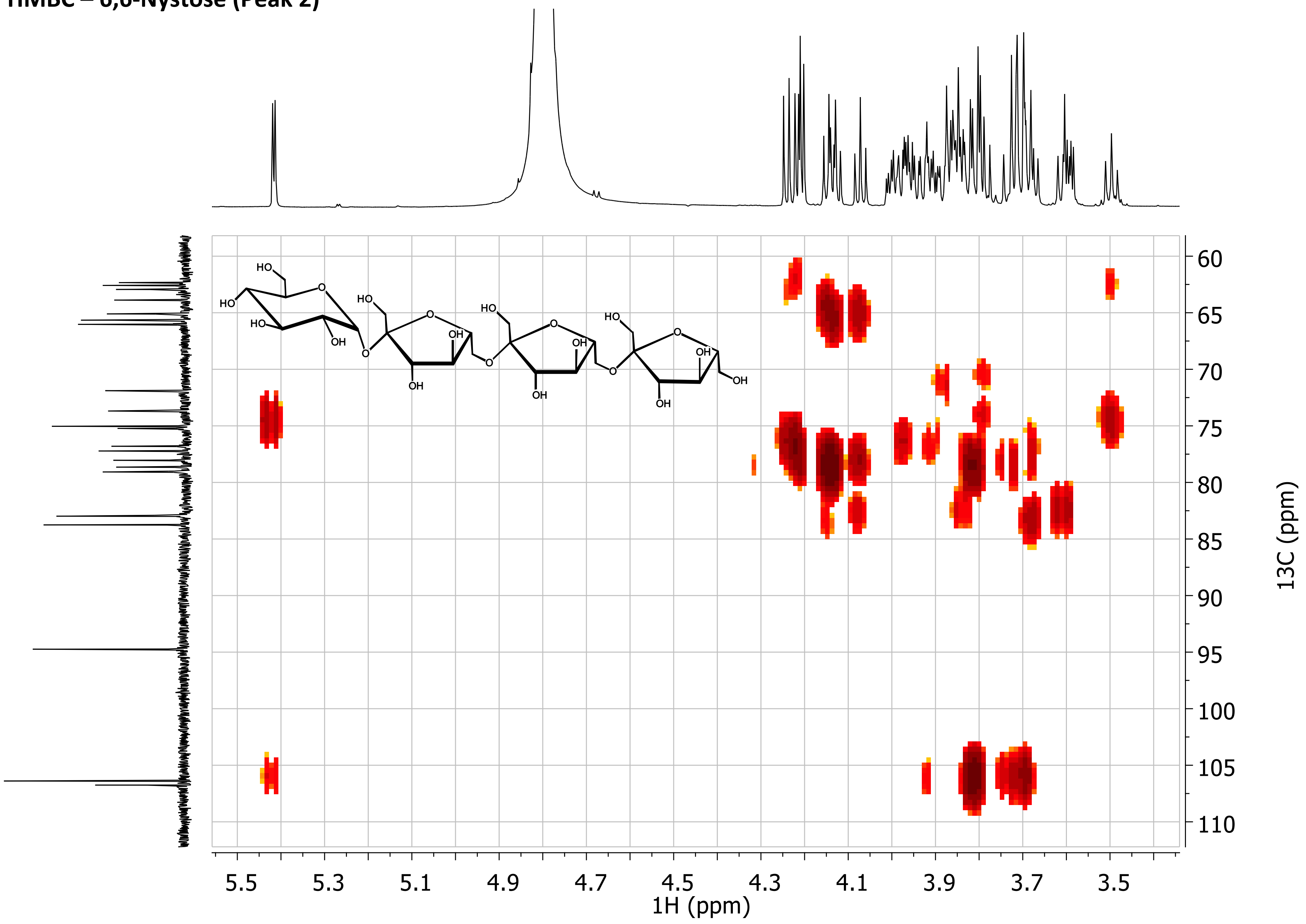

1H – 6-neo-Nystose (Peak 3)

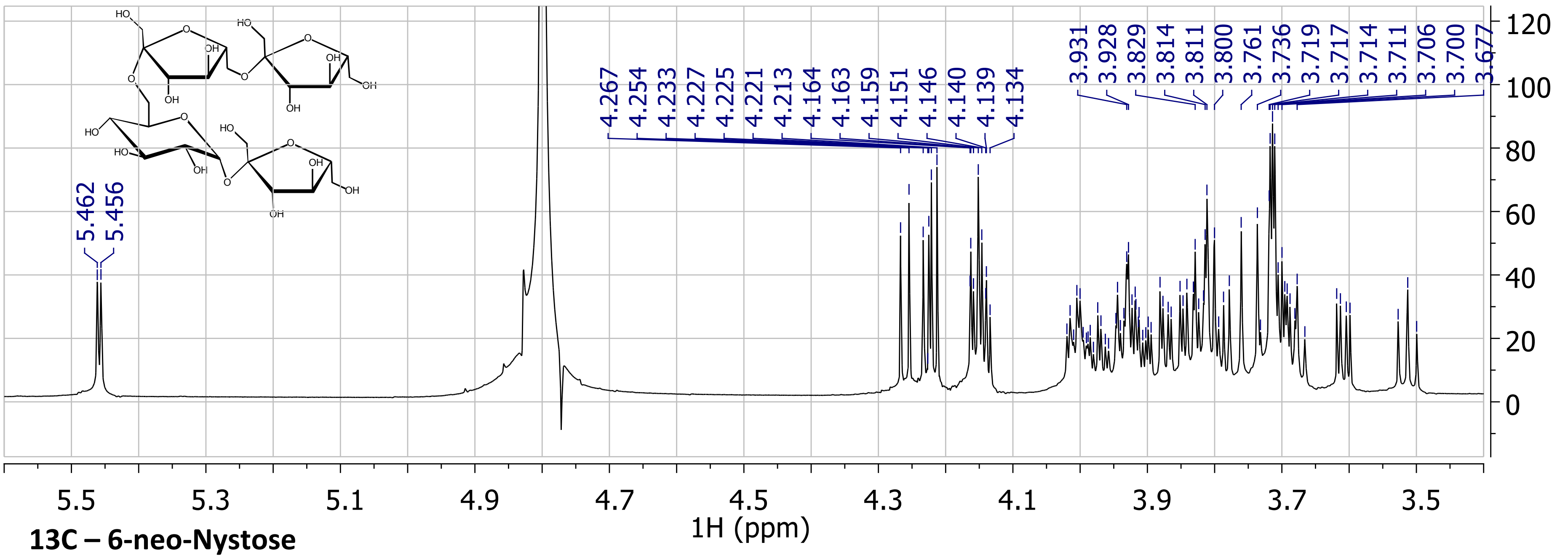

13C – 6-neo-Nystose

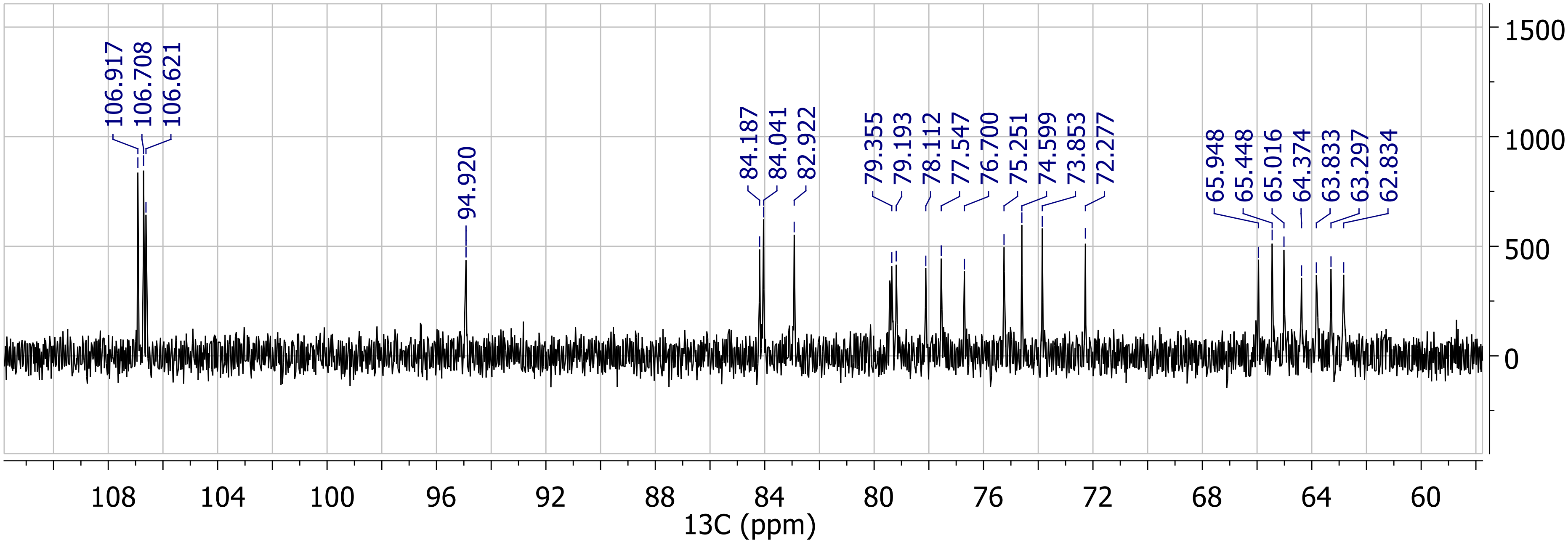

HSQC – 6-neo-Nystose (Peak 3)

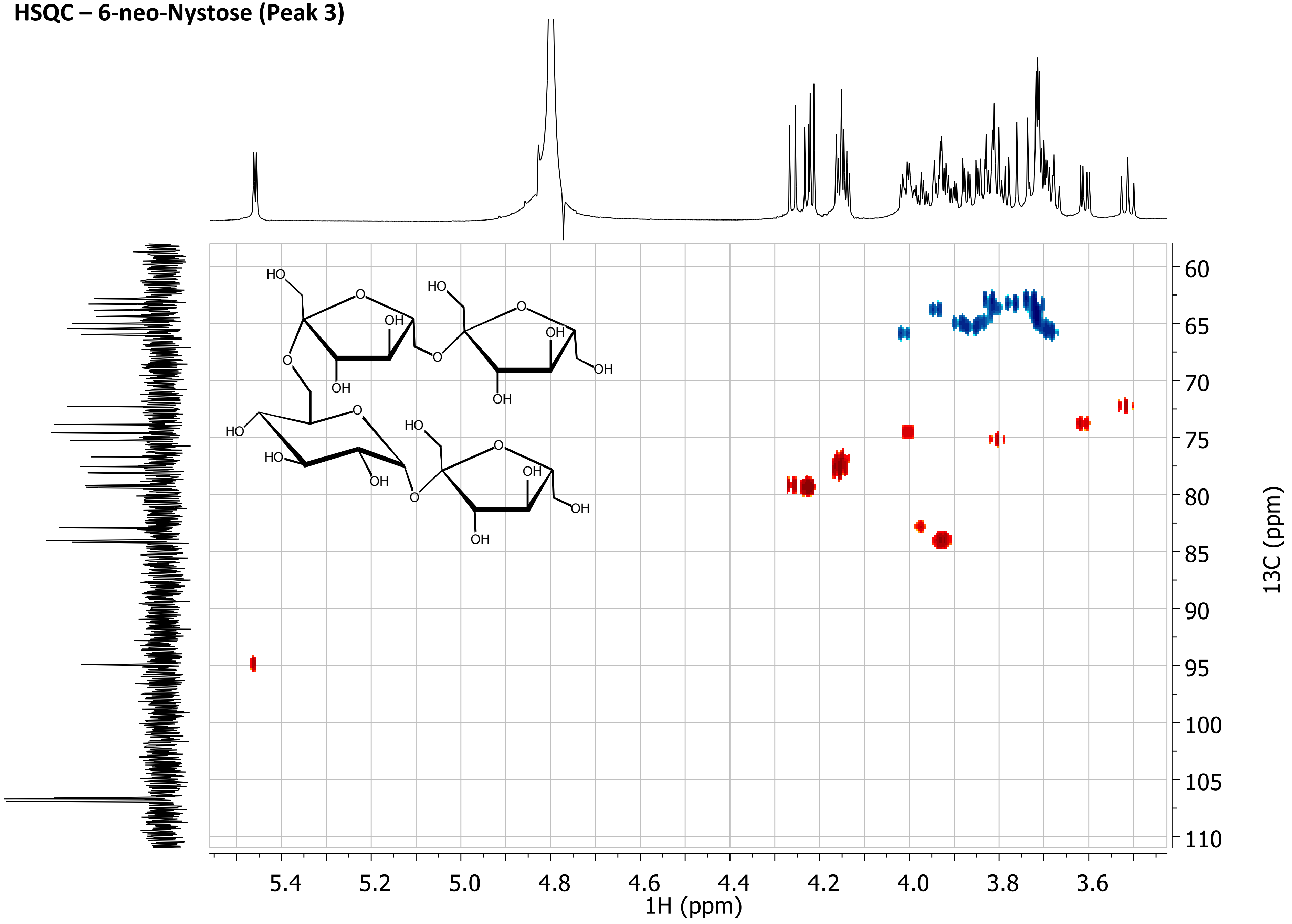

COSY – 6-neo-Nystose (Peak 3)

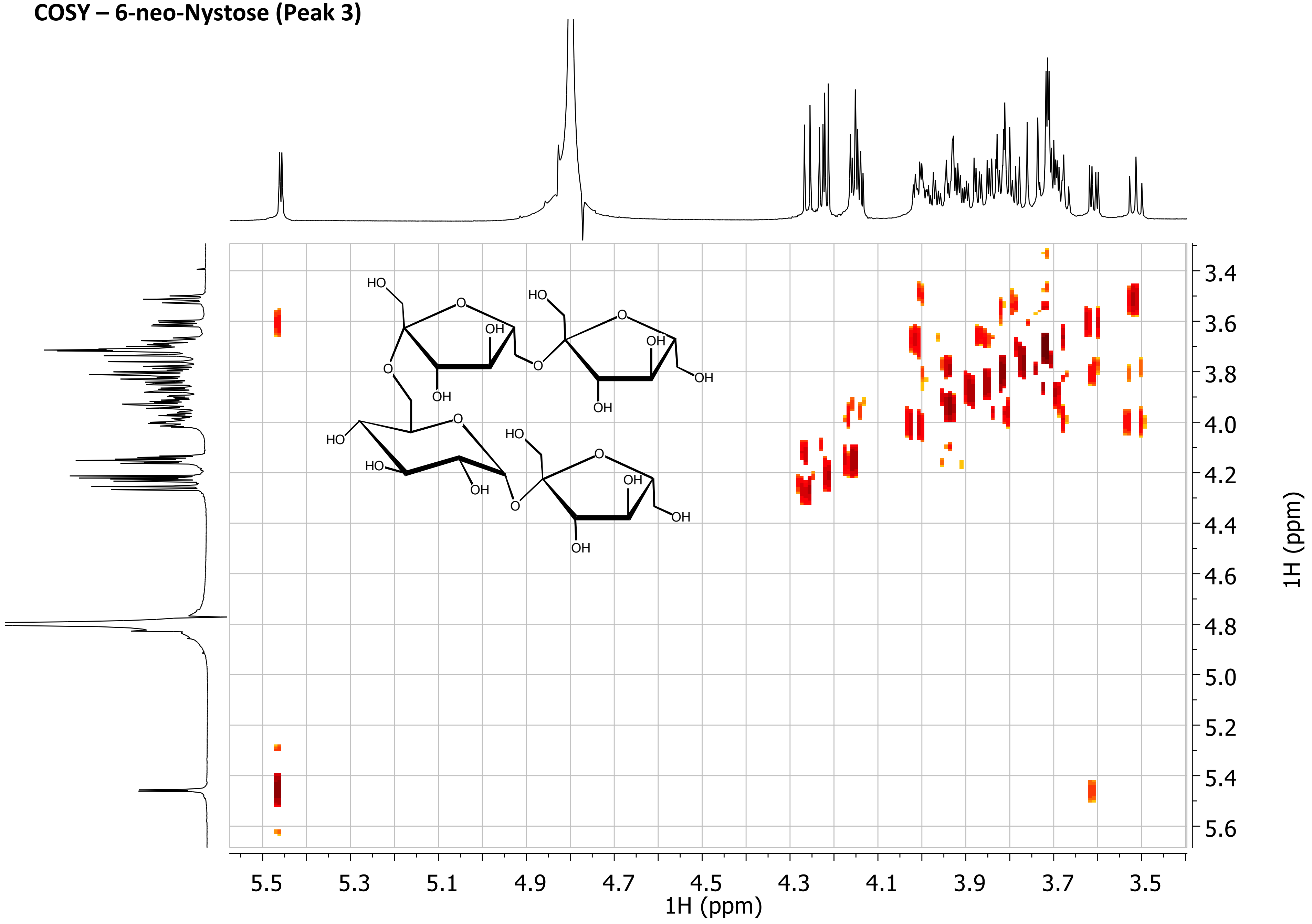

HMBC – 6-neo-Nystose (Peak 3)

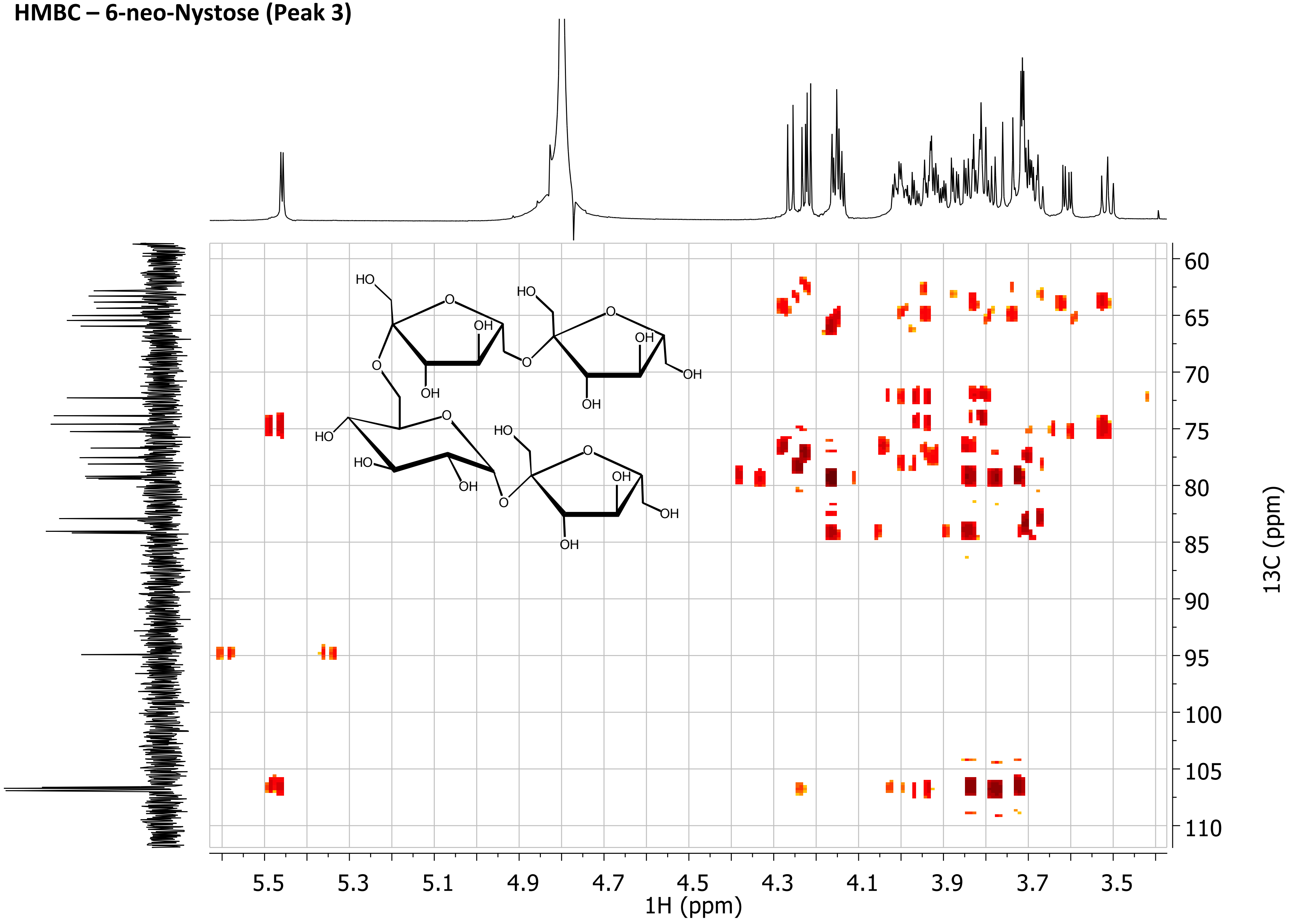

1H – Ercose (Peak 1’)

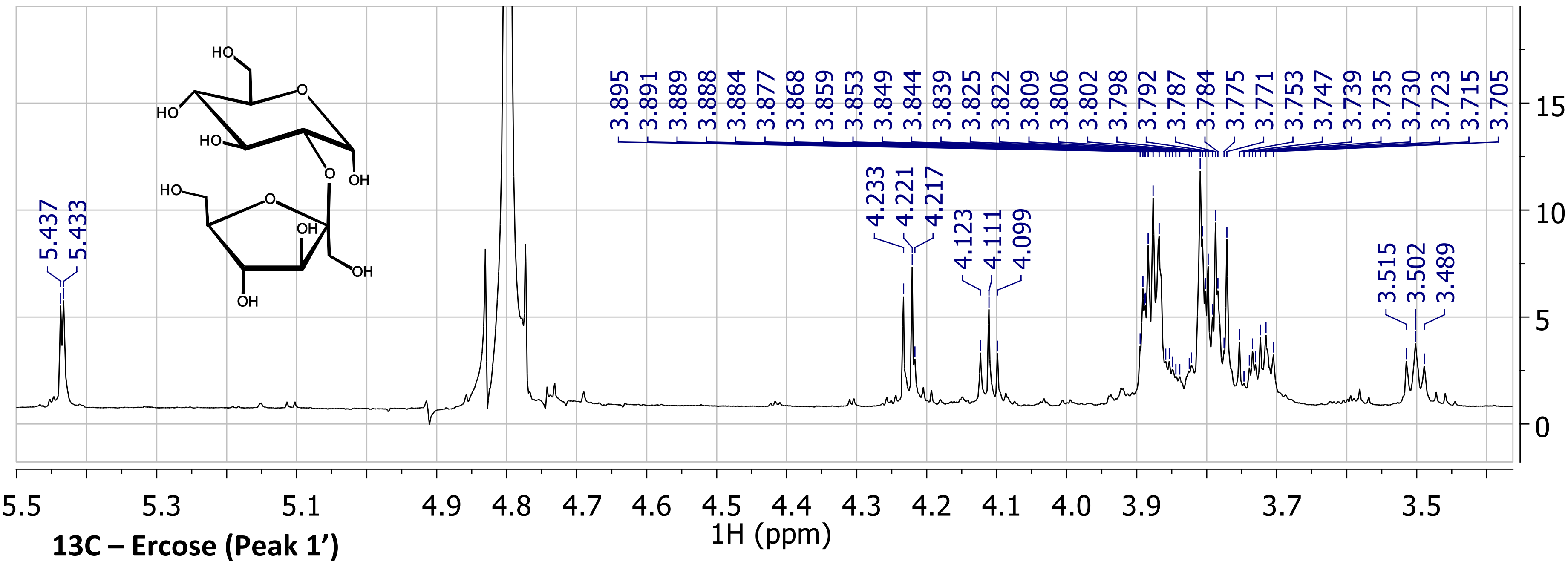

13C – Ercose (Peak 1’)

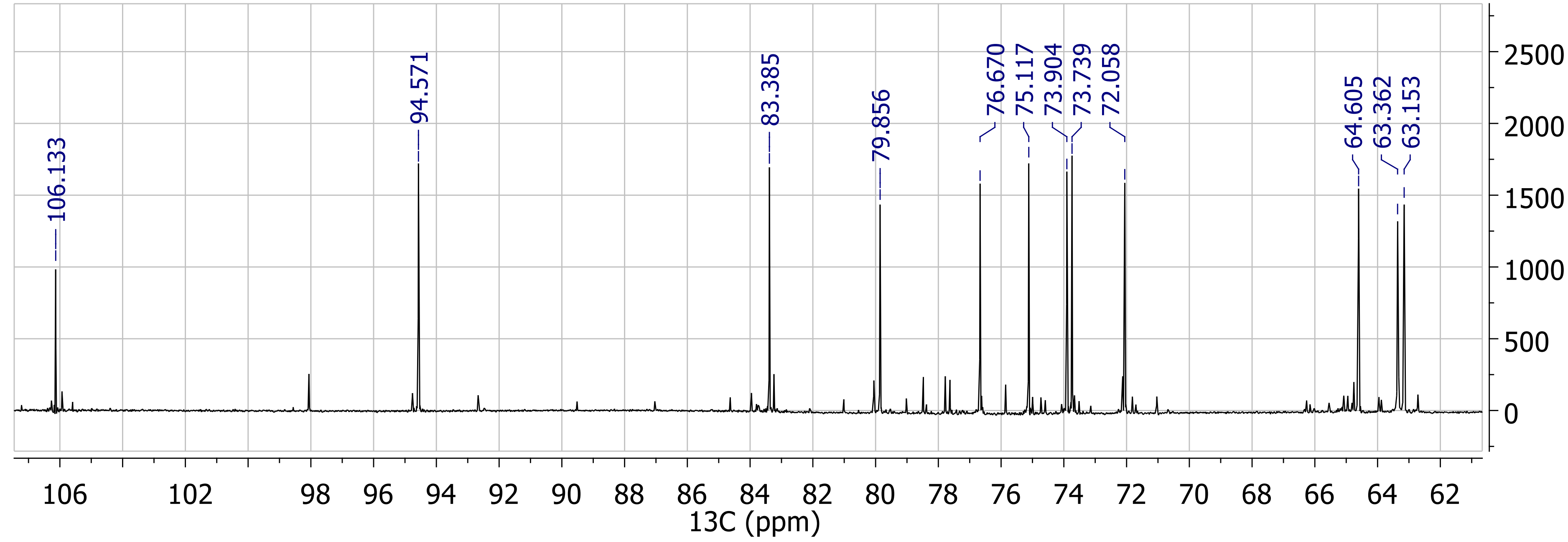

HSQC – Ercose (Peak 1’)

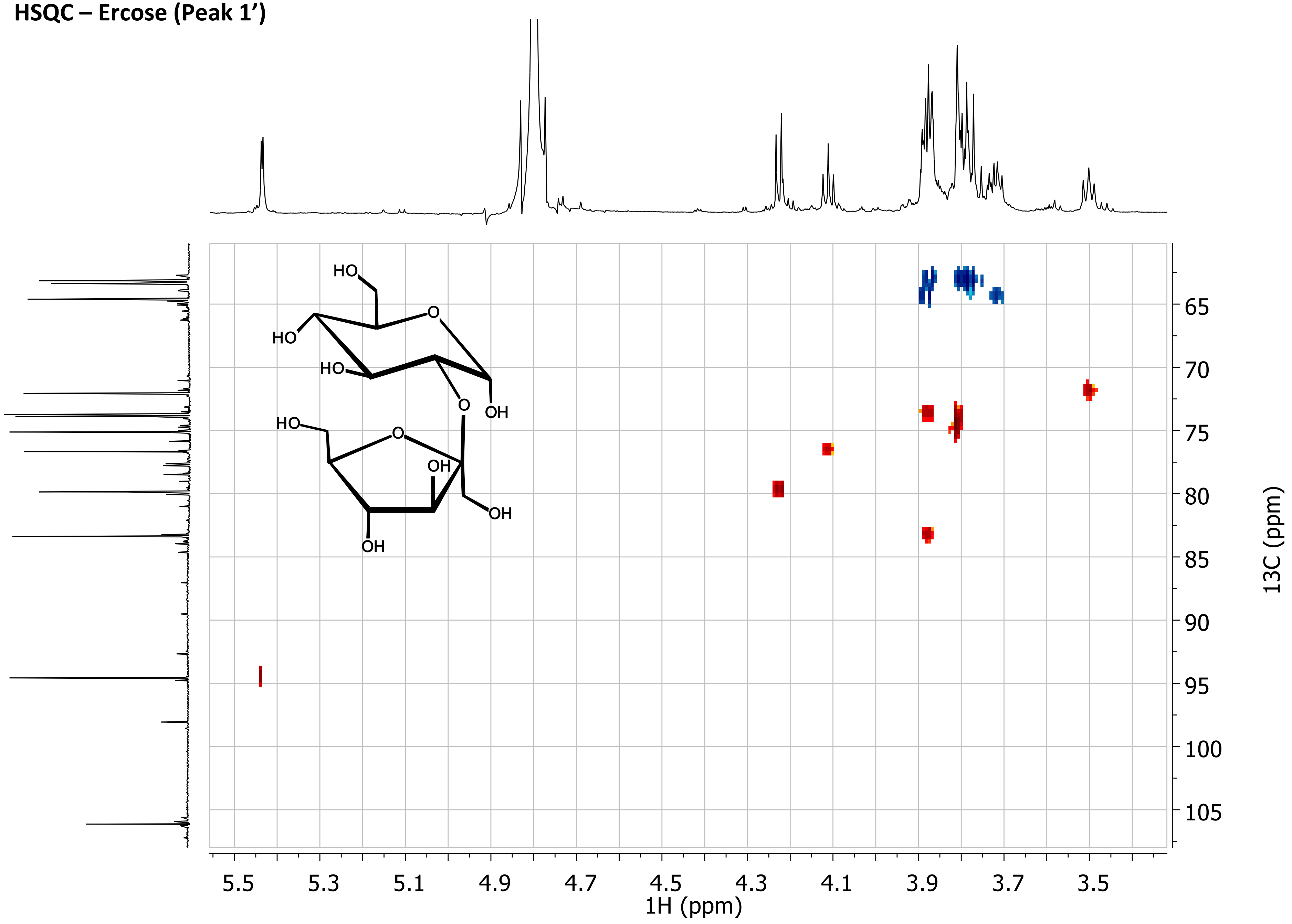

COSY – Ercose (Peak 1')

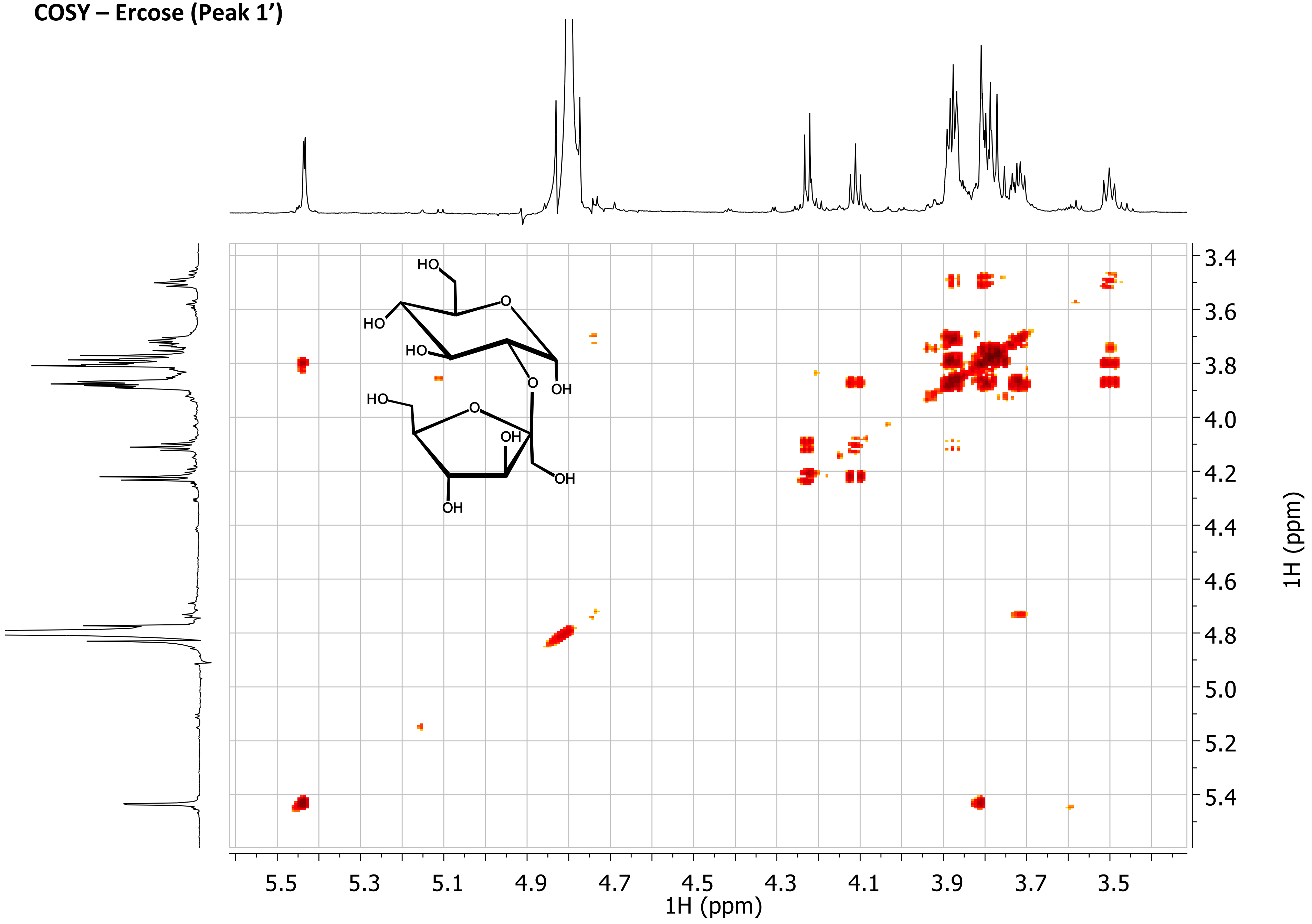

HMBC – Ercose (Peak 1’)

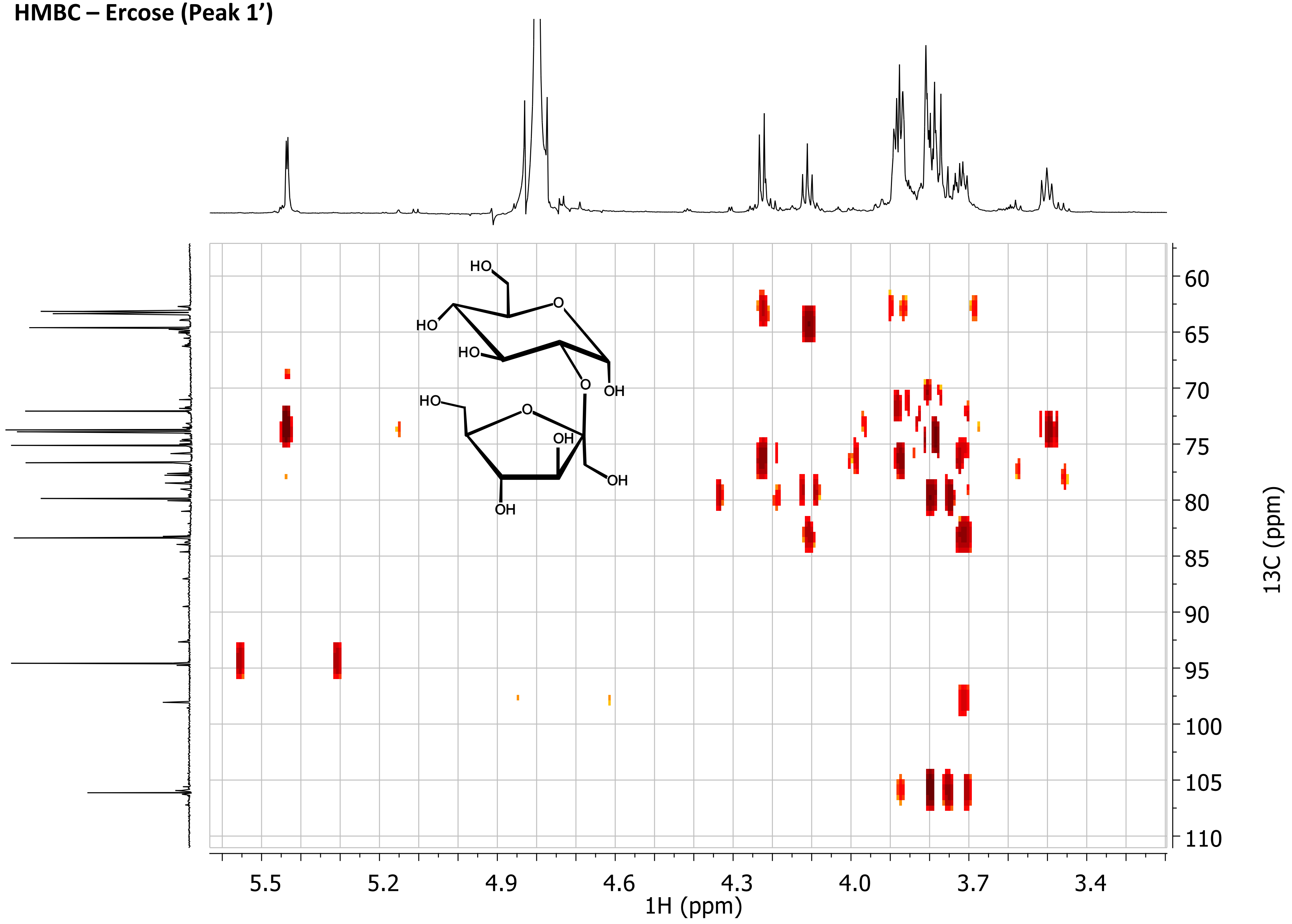

1H – α/β-Blastotriose (Peak 2')

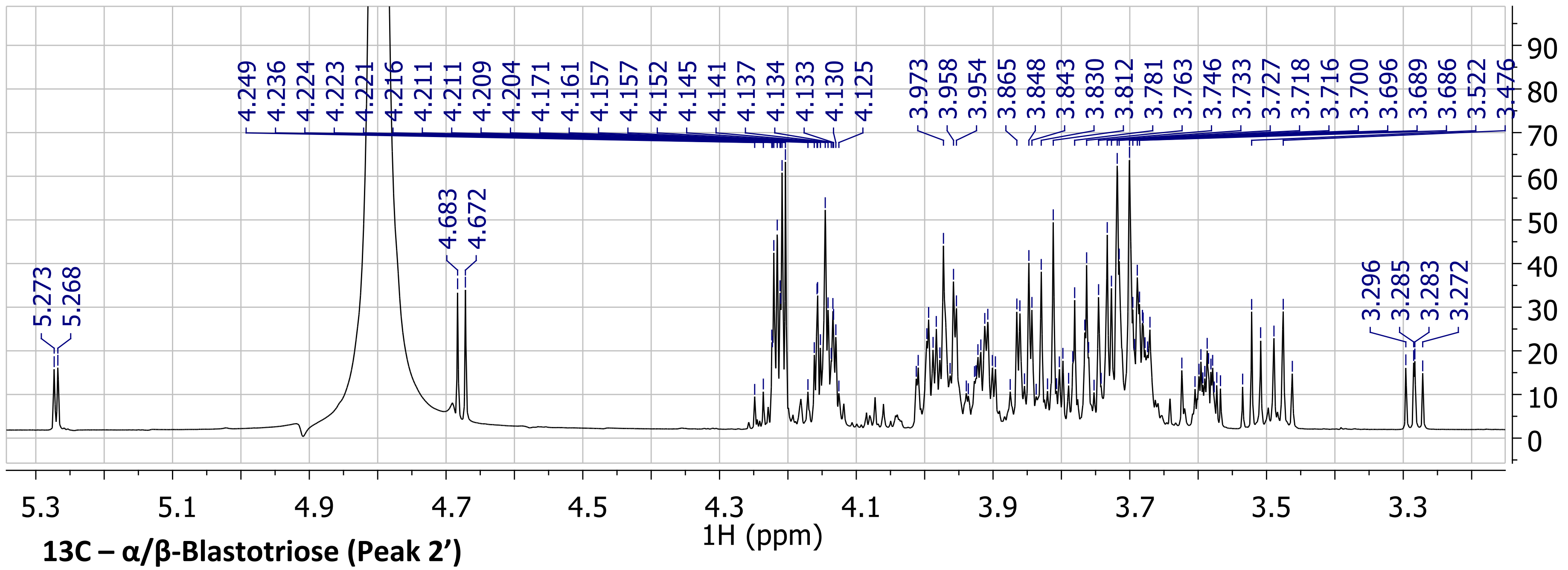

13C – α/β-Blastotriose (Peak 2')

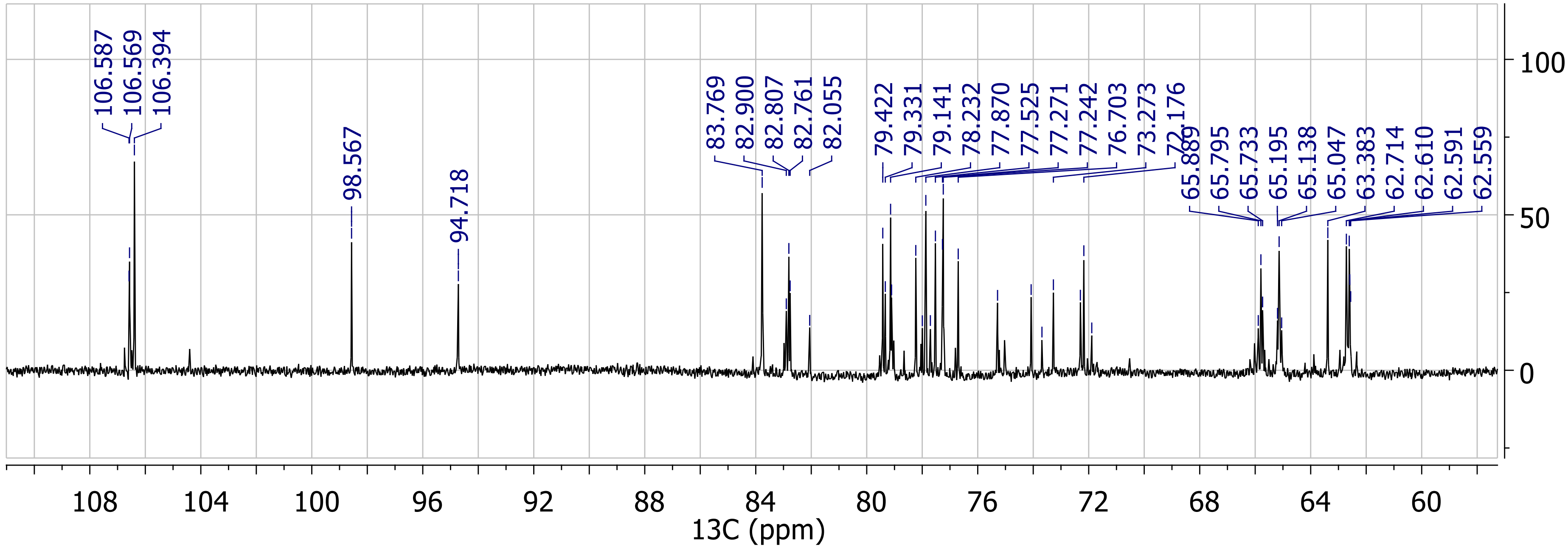

HSQC – α/β-Blastotriose (Peak 2')

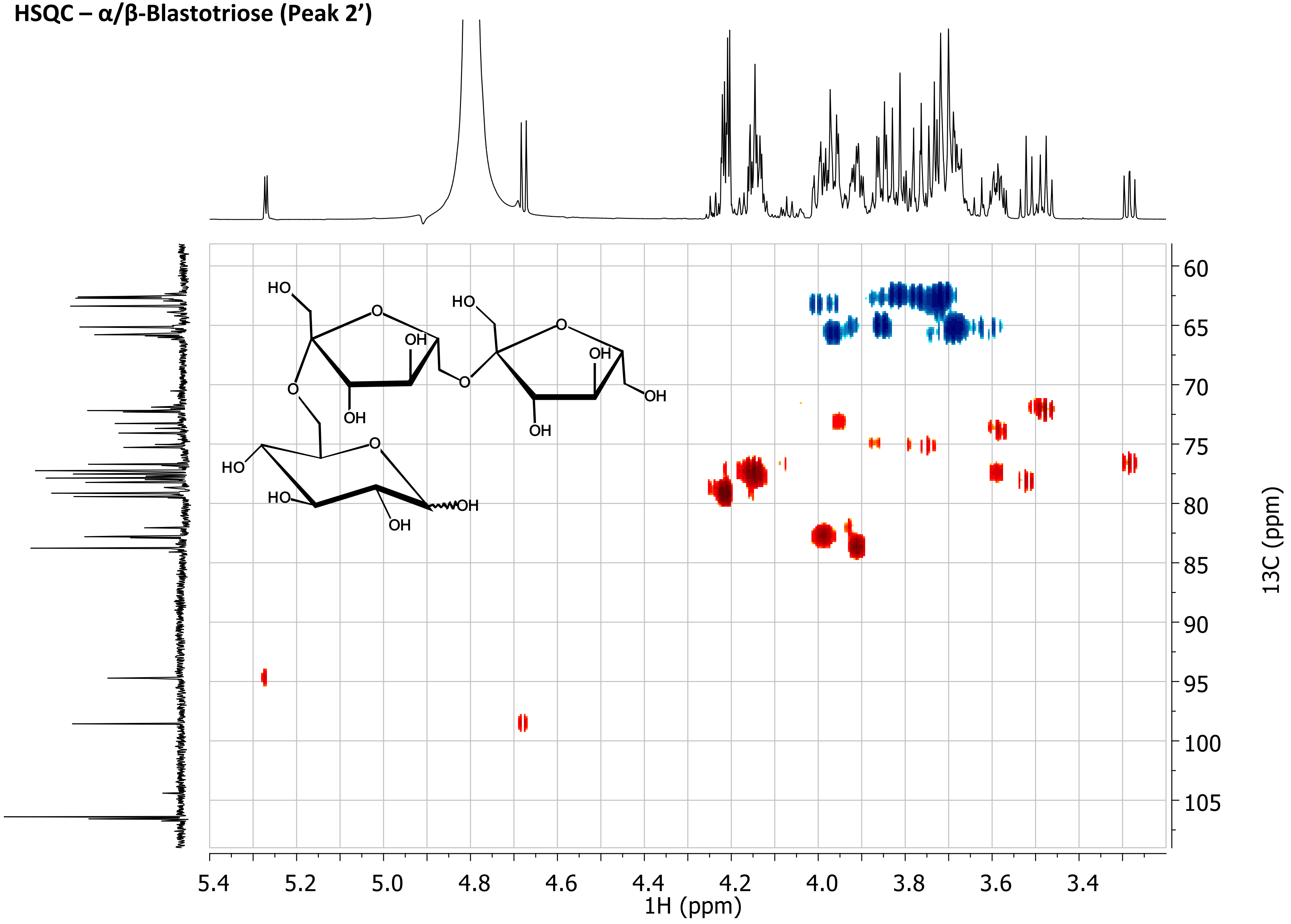

COSY – α/β-Blastotriose (Peak 2')

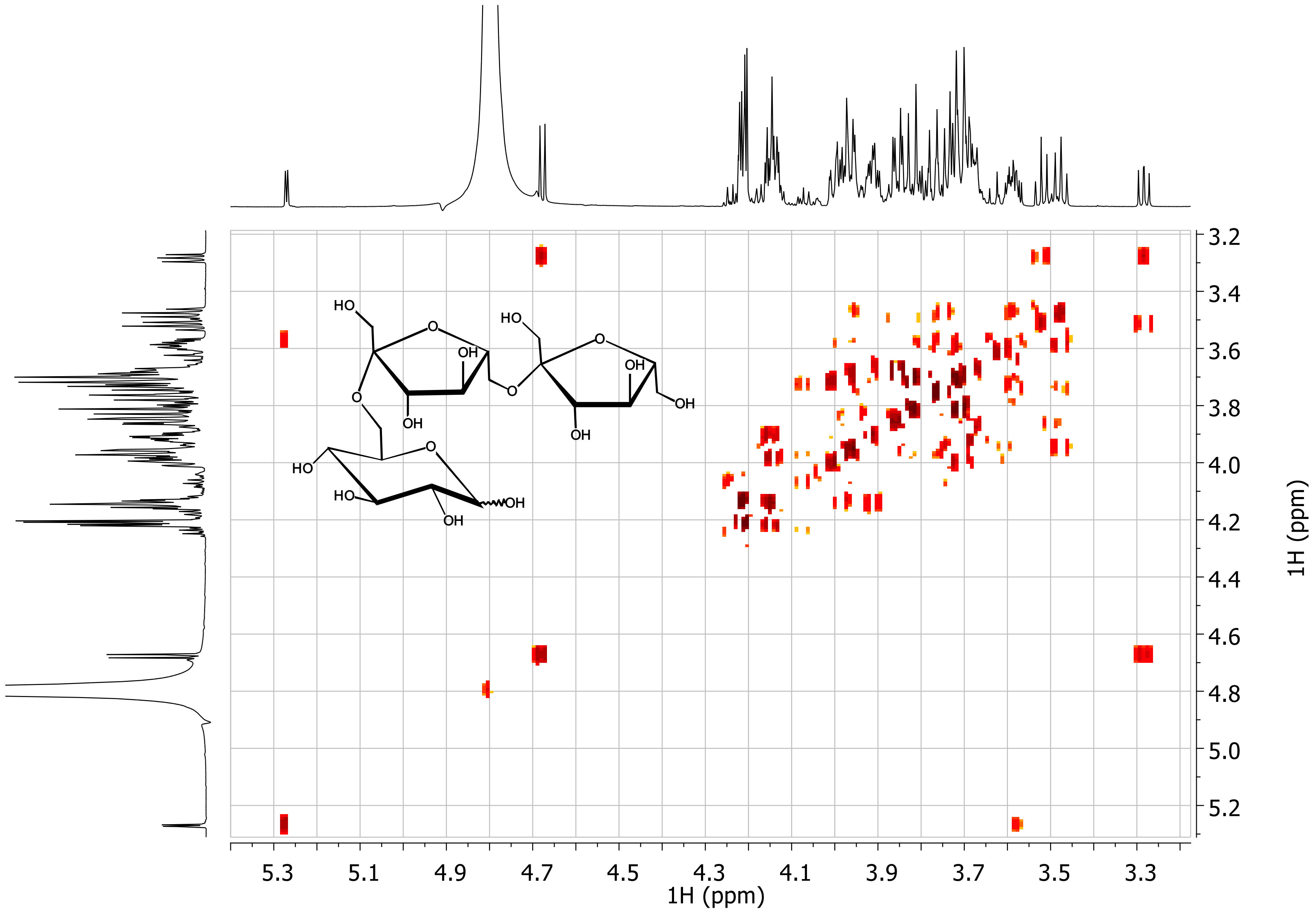

HMBC – α/β-Blastotriose (Peak 2’)

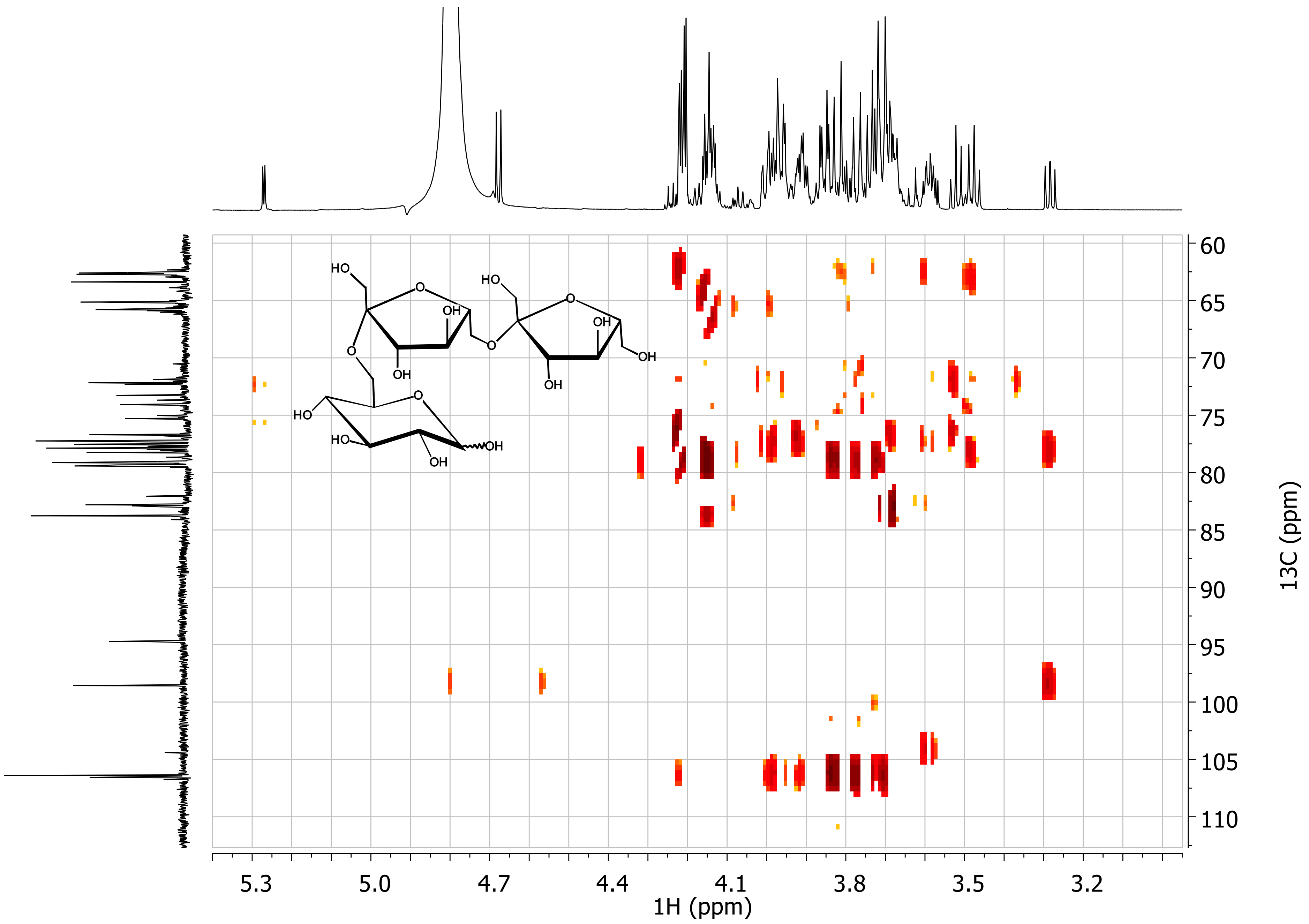

1H – Levantriose (Peak 4’)

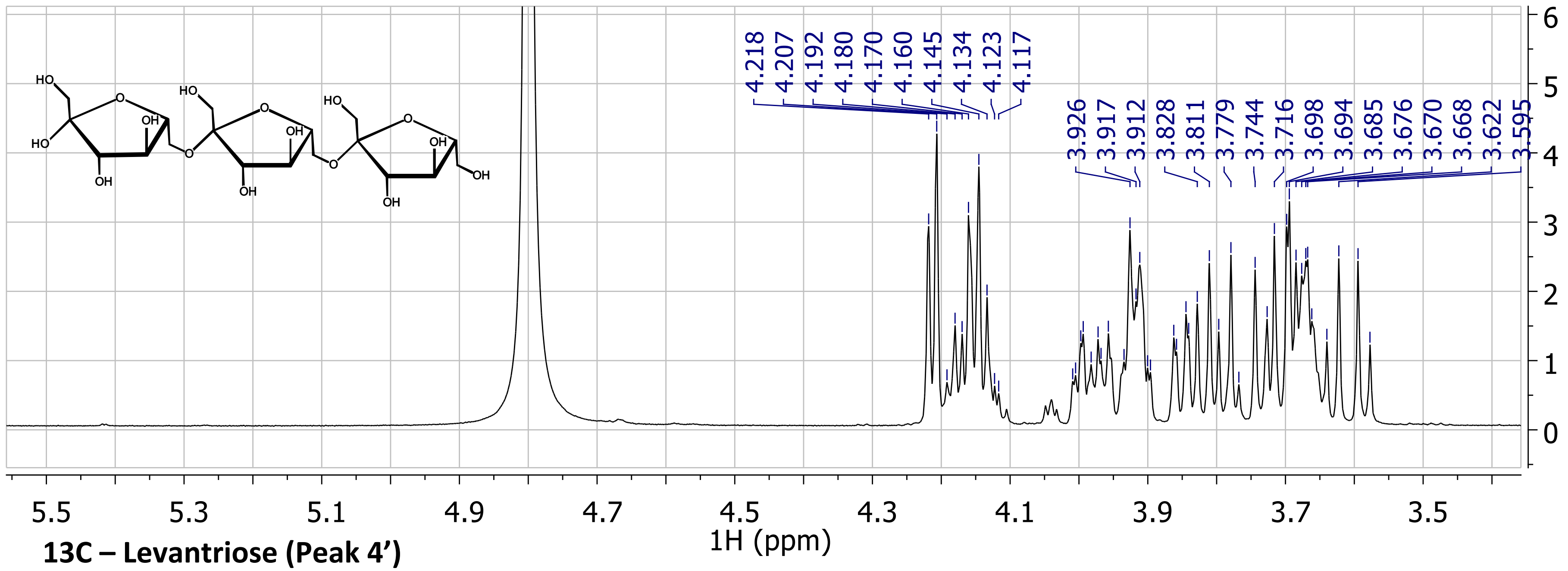

13C – Levantriose (Peak 4’)

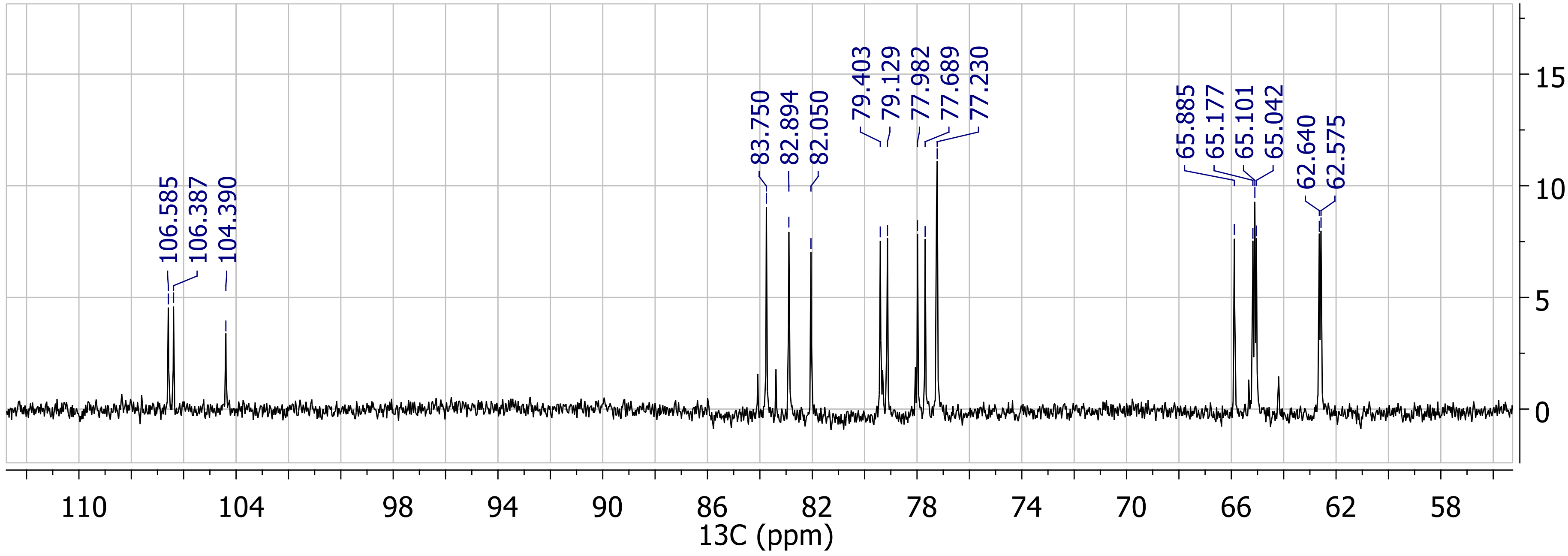

HSQC – Levantriose (Peak 4')

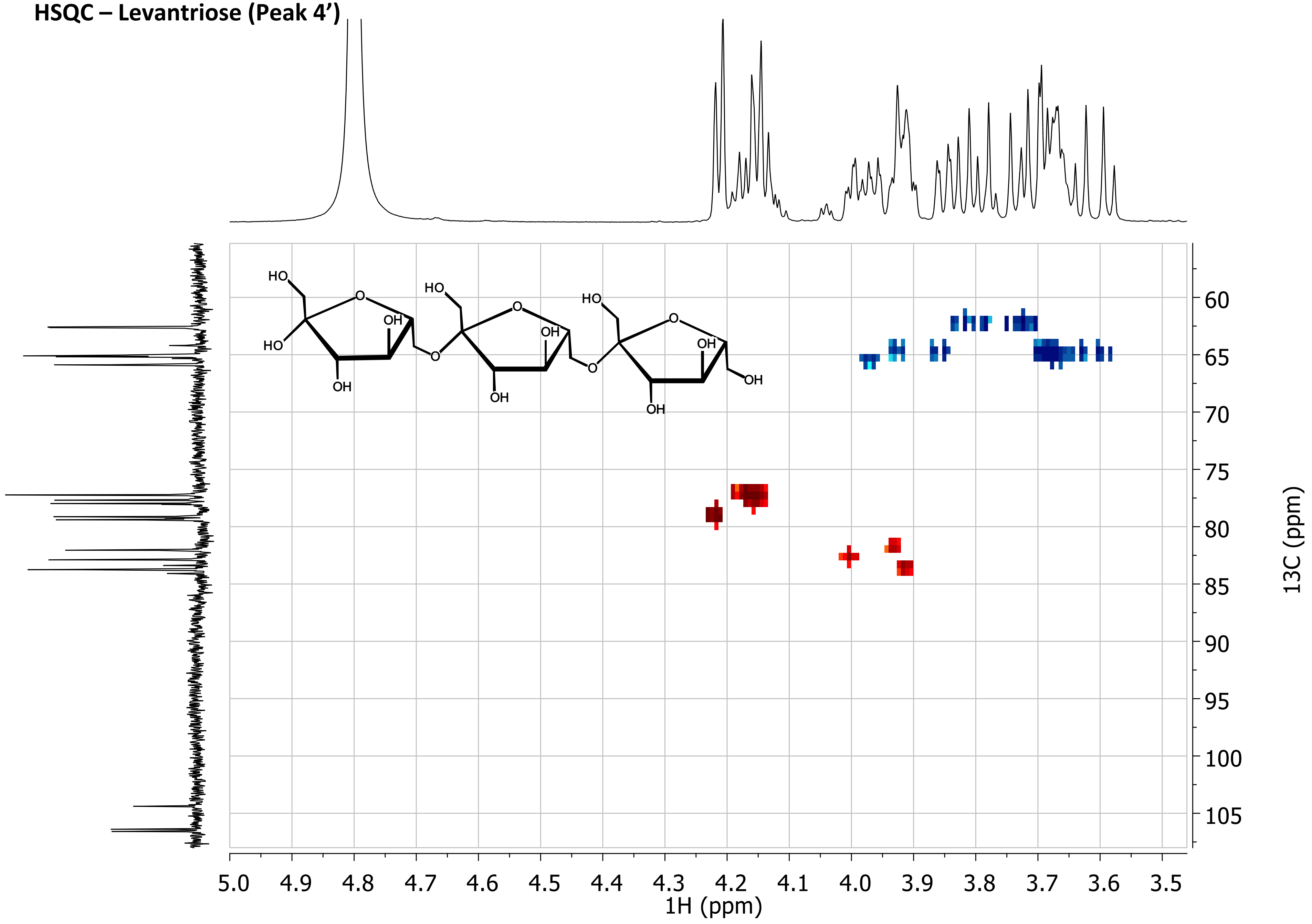

COSY – Levantriose (Peak 4')

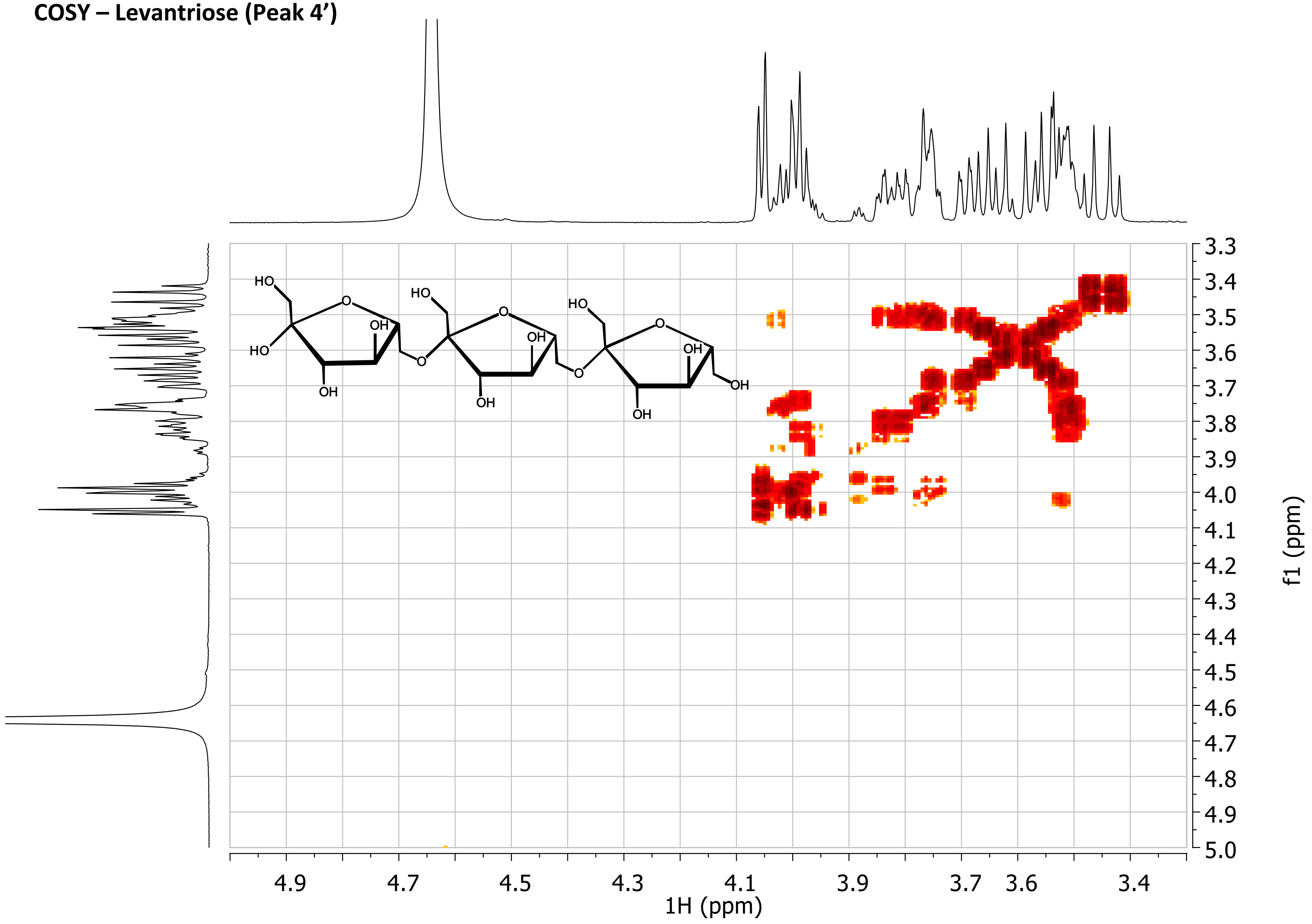

HMBC – Levantriose (Peak 4')

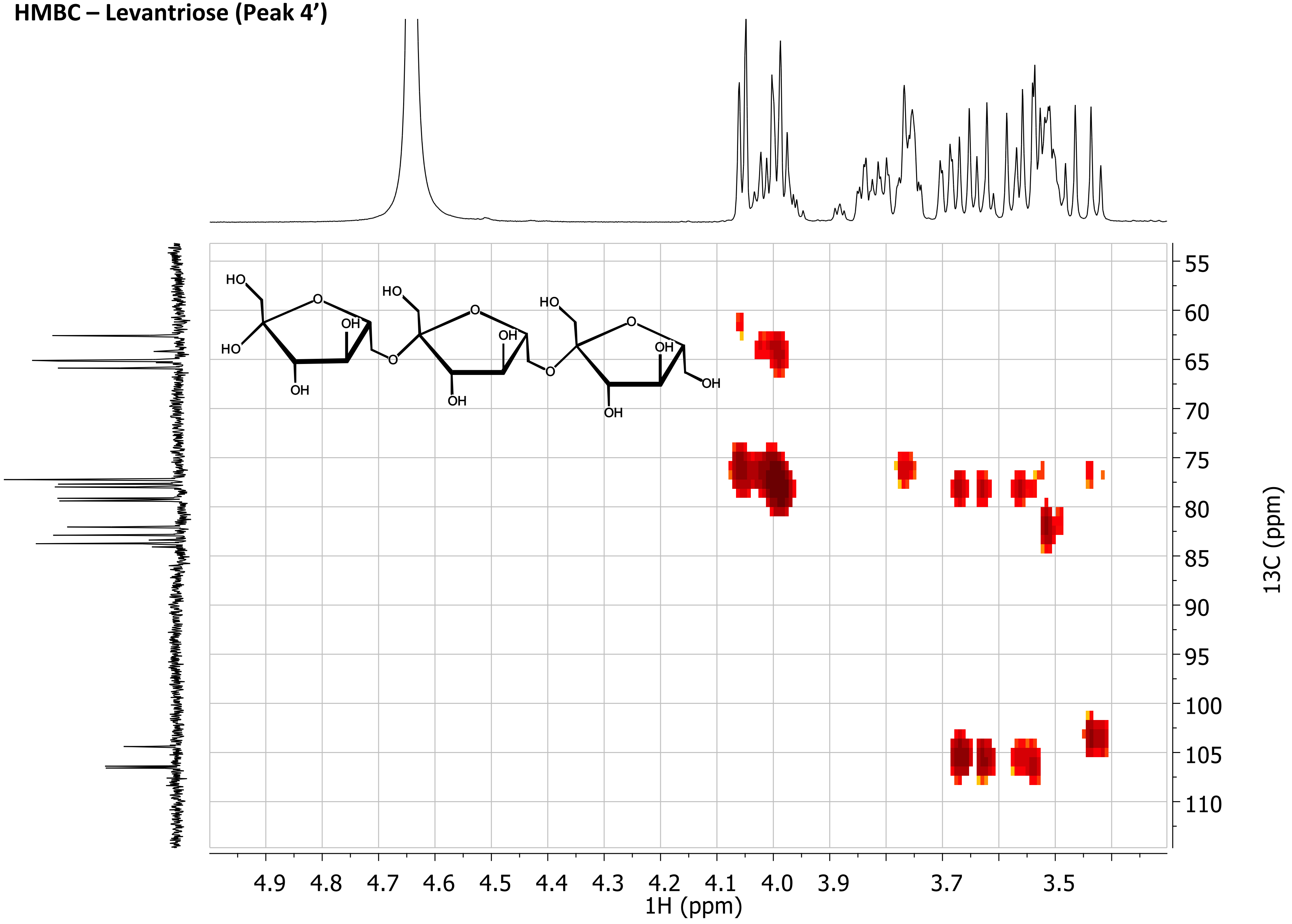

Supplement: Supplementary file 1 — Supplementary Figures and Information [file 41598_2018_32872_MOESM1_ESM.pdf]
